# Supplementary figures and images for: mdciao: Accessible Analysis and Visualization of Molecular Dynamics Simulation Data
Source: PLoS Comput Biol. 2025 Apr 21;21(4):e1012837. doi: 10.1371/journal.pcbi.1012837 (PMC12011235; doi:10.1371/journal.pcbi.1012837)

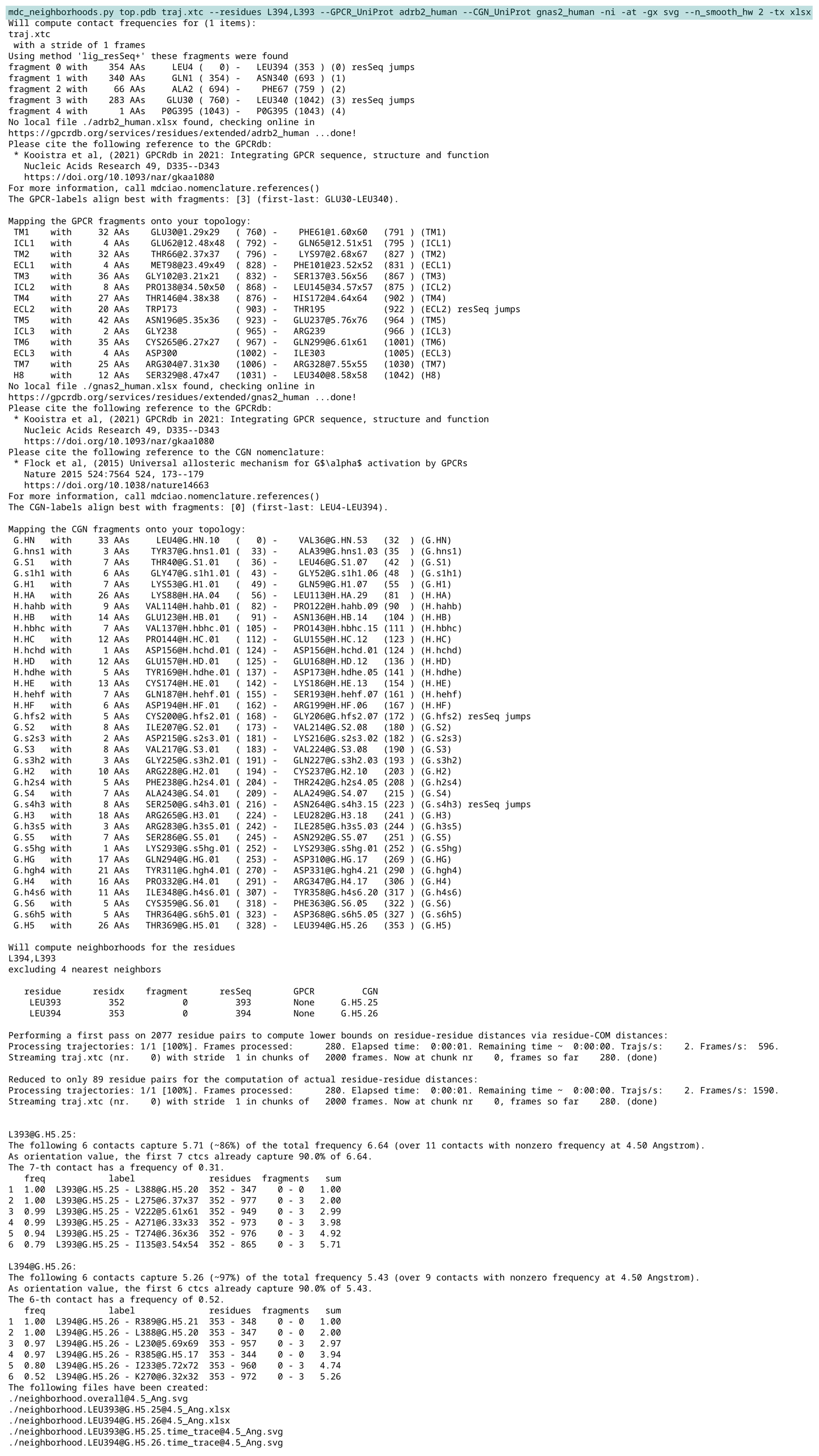

Supplement: S1 Fig — (TIF) [file pcbi.1012837.s001.tif]

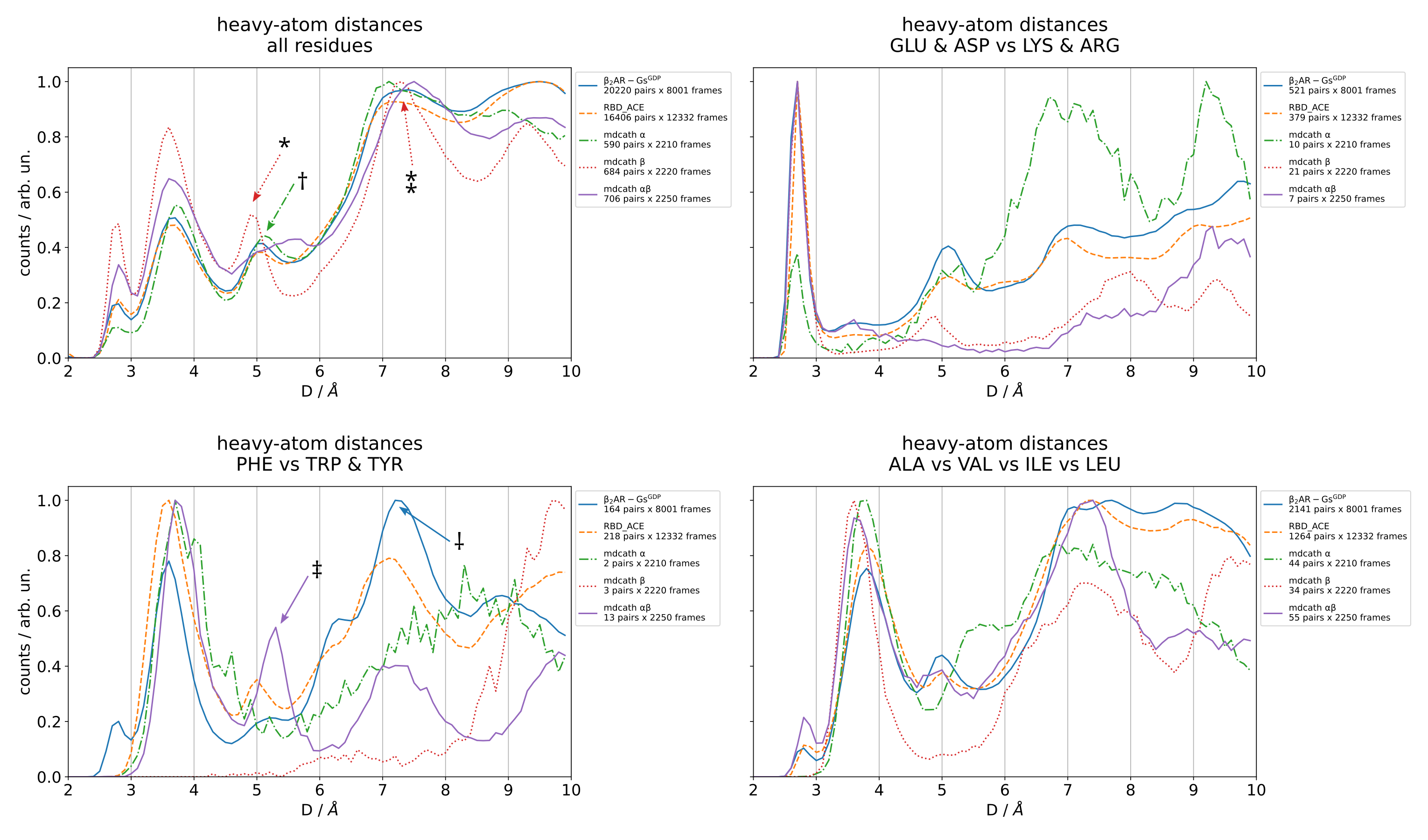

Supplement: S2 Fig — The distributions are shown for all residue-pair types in a), and then for specific residue types: b) residues with charged sidechains, c) residues with aromatic sidechains, discarding the polar OH-group in PHE and d) residues with nonpolar sidechains. These simulations have been carried out with different (but comparable) simulation engines, parameters and forcefields, but roughly recapture each other’s peak-positions, with slight shifts along the x-axis. Selected, longer-range peaks denoted with *, ⁑, †, ‡, and ⸸ are shown in S4 Fig via snapshots of the trajectory. (TIF) [file pcbi.1012837.s002.tif]

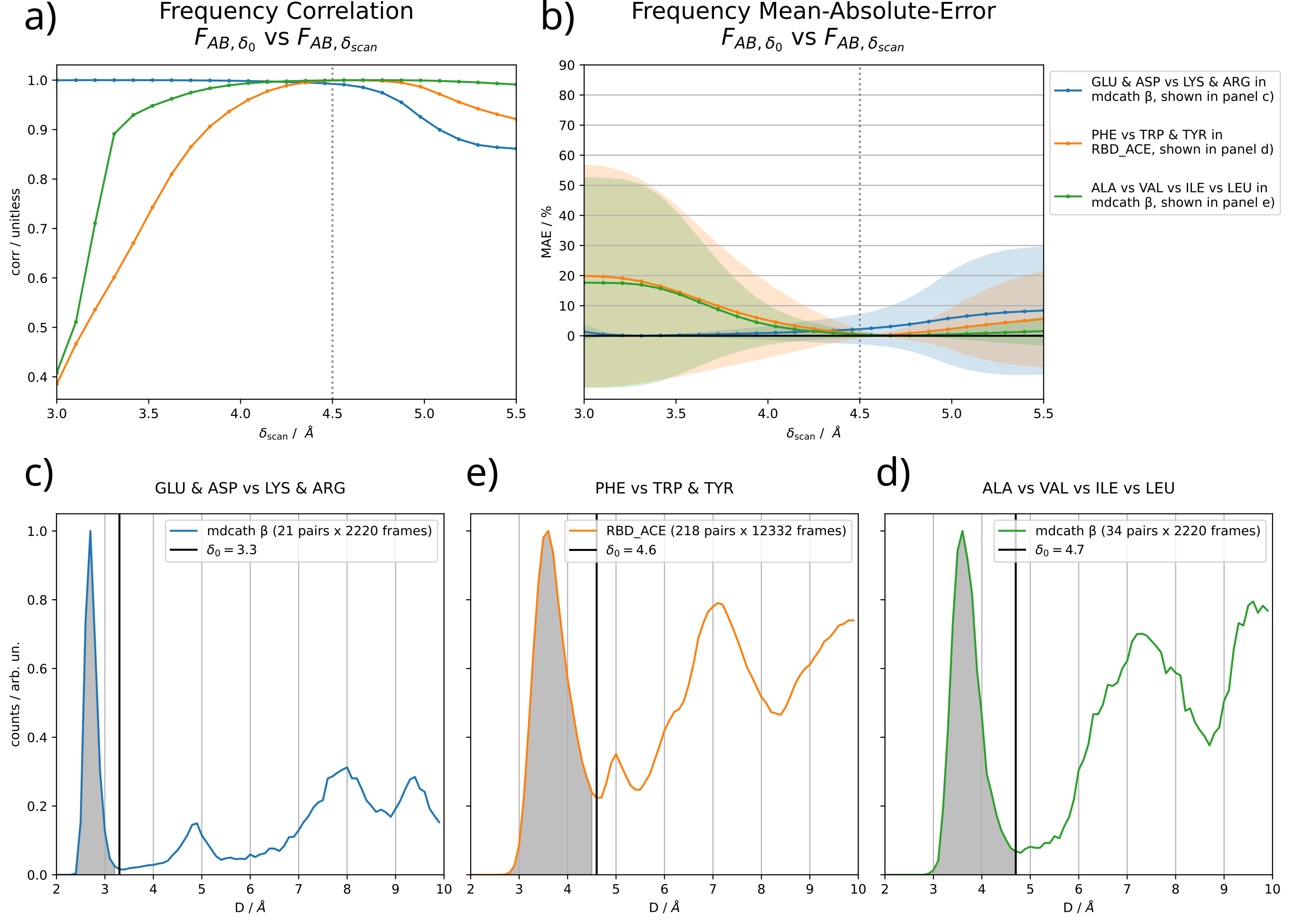

Supplement: S3 Fig — The peaks have different centers and shapes depending on the interaction type and on the dataset used, hence for this sample we have chosen representative distributions in which only one peak is captured (gray area under the curves). Panel a) shows the high (>.95) correlations around δscan between 4-5 Å. We have included the default value of mdciao δ = 4.5 Å as a dotted vertical line. Panel b) shows the mean-absolute-error (MAE) in the frequencies, in absolute percentage terms. Around 4.5Å, the MAE is less than 5% (solid lines) and standard deviation is around that value (the 95% confidence is shown as shaded area). (TIF) [file pcbi.1012837.s003.tif]

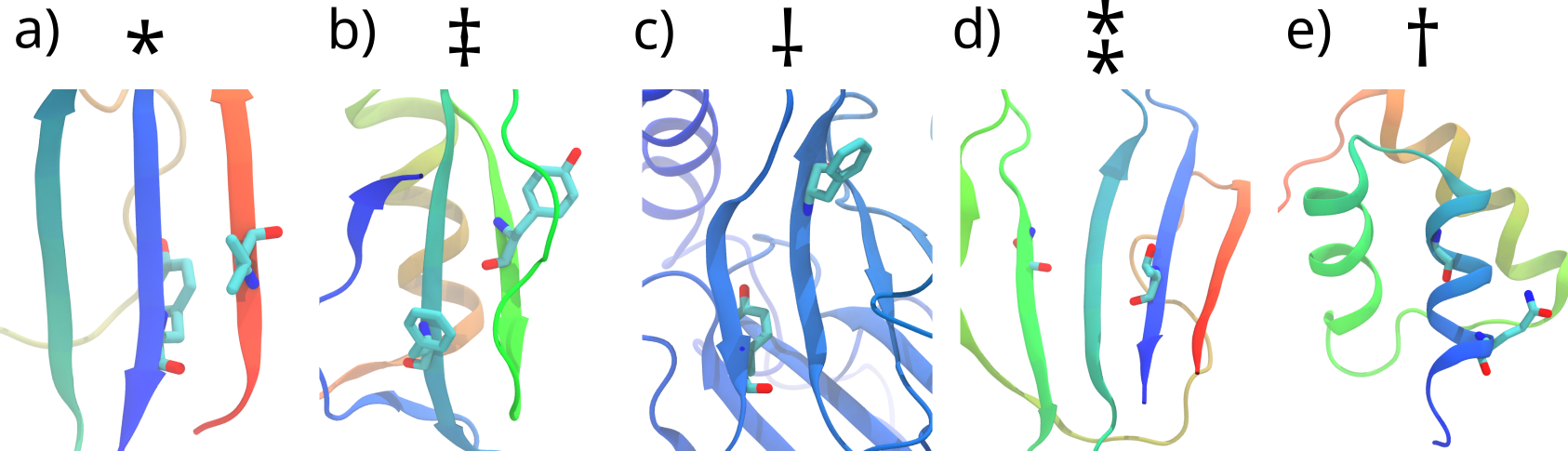

Supplement: S4 Fig — Residue pairs like the ones shown in panels a), b), c) and d) are in the vicinity of each other by virtue of sharing beta-sheet structure, but are offset with respect to the actual backbone-backbone hydrogen-bond interaction. e) denotes residue pairs corresponding to the fifth residue after a full alpha-helical turn. (TIF) [file pcbi.1012837.s004.tif]
