# Supplementary material for: mdciao: Accessible Analysis and Visualization of Molecular Dynamics Simulation Data
Source: PLoS Comput Biol. 2025 Apr 21;21(4):e1012837. doi: 10.1371/journal.pcbi.1012837 (PMC12011235; doi:10.1371/journal.pcbi.1012837)
Supplement: S1 Notebook — (PDF) [file pcbi.1012837.s007.pdf]

Interface of β2 Adrenergic Receptor in Complex with Empty Gs-Protein

In [1]:
```
import mdtraj as md
import mdciao
```

Download example data and load into the namespace

In [2]:
```
import numpy as np
import os
if not os.path.exists("mdciao_example"):
    mdciao.examples.fetch_example_data("b2ar@Gs")
traj = md.load("mdciao_example/traj.xtc",top="mdciao_example/top.pdb")
```

```
Unzipping to 'mdciao_example'
```

Create consensus labeler objects

In [3]:
```
GPCR = mdciao.nomenclature.LabelerGPCR("adrb2_human")
CGN = mdciao.nomenclature.LabelerCGN("GNAS2_HUMAN")
```

```
No local file ./adrb2_human.xlsx found, checking online in
https://gpcrdb.org/services/residues/extended/adrb2_human ...done!
Please cite the following reference to the GPCRdb:
 * Kooistra et al, (2021) GPCRdb in 2021: Integrating GPCR sequence, structure and function
   Nucleic Acids Research 49, D335--D343
   https://doi.org/10.1093/nar/gkaa1080
For more information, call mdciao.nomenclature.references()
No local file ./GNAS2_HUMAN.xlsx found, checking online in
https://gpcrdb.org/services/residues/extended/gnas2_human ...done!
Please cite the following reference to the GPCRdb:
 * Kooistra et al, (2021) GPCRdb in 2021: Integrating GPCR sequence, structure and function
   Nucleic Acids Research 49, D335--D343
   https://doi.org/10.1093/nar/gkaa1080
Please cite the following reference to the CGN nomenclature:
 * Flock et al, (2015) Universal allosteric mechanism for G$\alpha$ activation by GPCRs
   Nature 2015 524:7564 524, 173--179
   https://doi.org/10.1038/nature14663
For more information, call mdciao.nomenclature.references()
```

Guess molecular fragments

This would be done anyway by the `mdciao.cli.interface` call in the cell below, here we do it have the fragments defined in the namespace

In [4]:
```
fragments = mdciao.fragments.get_fragments(traj.top);
fragment_names = ["Galpha", "Gbeta", "Ggamma","B2AR","P0G"]
```

```
Auto-detected fragments with method 'lig_resSeq+'
fragment      0 with    354 AAs     LEU4 (    0) -   LEU394 (353 ) (0) resSeq jumps
fragment      1 with    340 AAs     GLN1 ( 354) -   ASN340 (693 ) (1)
fragment      2 with     66 AAs     ALA2 ( 694) -    PHE67 (759 ) (2)
fragment      3 with    283 AAs    GLU30 ( 760) -   LEU340 (1042) (3) resSeq jumps
fragment      4 with      1 AAs   P0G395 (1043) -   P0G395 (1043) (4)
```

Compute Gα-B2AR interface

Using the above fragment definitions

In [5]:
```
intf = mdciao.cli.interface(traj,
                            title="3SN6 beta2AR-Galpha interface",
                            fragments=fragments, fragment_names = fragment_names,
                            interface_selection_1=[0],
                            interface_selection_2=[3],
                            GPCR_UniProt=GPCR, CGN_UniProt=CGN,
                            accept_guess=True, no_disk=True, figures=False)
```

```
Will compute contact frequencies for trajectories:
<mdtraj.Trajectory with 280 frames, 8384 atoms, 1044 residues, and unitcells>
 with a stride of 1 frames
Using method 'user input by residue array or range' these fragments were found
fragment Galpha with    354 AAs     LEU4 (   0) -   LEU394 (353 ) (Galpha) resSeq jumps
fragment Gbeta  with    340 AAs     GLN1 ( 354) -   ASN340 (693 ) (Gbeta)
fragment Ggamma with     66 AAs     ALA2 ( 694) -    PHE67 (759 ) (Ggamma)
fragment B2AR   with    283 AAs    GLU30 ( 760) -   LEU340 (1042) (B2AR) resSeq jumps
fragment P0G    with      1 AAs   P0G395 (1043) -   P0G395 (1043) (P0G)
The GPCR-labels align best with fragments: [3] (first-last: GLU30-LEU340).

Mapping the GPCR fragments onto your topology:
 TM1    with     32 AAs    GLU30@1.29x29   ( 760) -    PHE61@1.60x60   (791 ) (TM1)
 ICL1   with      4 AAs    GLU62@12.48x48  ( 792) -    GLN65@12.51x51  (795 ) (ICL1)
 TM2    with     32 AAs    THR66@2.37x37   ( 796) -    LYS97@2.68x67   (827 ) (TM2)
 ECL1   with      4 AAs    MET98@23.49x49  ( 828) -   PHE101@23.52x52  (831 ) (ECL1)
 TM3    with     36 AAs   GLY102@3.21x21   ( 832) -   SER137@3.56x56   (867 ) (TM3)
 ICL2   with      8 AAs   PRO138@34.50x50  ( 868) -   LEU145@34.57x57  (875 ) (ICL2)
 TM4    with     27 AAs   THR146@4.38x38   ( 876) -   HIS172@4.64x64   (902 ) (TM4)
 ECL2   with     20 AAs   TRP173           ( 903) -   THR195           (922 ) (ECL2) resSeq jumps
 TM5    with     42 AAs   ASN196@5.35x36   ( 923) -   GLU237@5.76x76   (964 ) (TM5)
 ICL3   with      2 AAs   GLY238           ( 965) -   ARG239           (966 ) (ICL3)
 TM6    with     35 AAs   CYS265@6.27x27   ( 967) -   GLN299@6.61x61   (1001) (TM6)
 ECL3   with      4 AAs   ASP300           (1002) -   ILE303           (1005) (ECL3)
 TM7    with     25 AAs   ARG304@7.31x30   (1006) -   ARG328@7.55x55   (1030) (TM7)
 H8     with     12 AAs   SER329@8.47x47   (1031) -   LEU340@8.58x58   (1042) (H8)
The CGN-labels align best with fragments: [0] (first-last: LEU4-LEU394).

Mapping the CGN fragments onto your topology:
 G.HN   with     33 AAs     LEU4@G.HN.10   (   0) -    VAL36@G.HN.53   (32  ) (G.HN)
 G.hns1 with      3 AAs    TYR37@G.hns1.01 (  33) -    ALA39@G.hns1.03 (35  ) (G.hns1)
 G.S1   with      7 AAs    THR40@G.S1.01   (  36) -    LEU46@G.S1.07   (42  ) (G.S1)
 G.s1h1 with      6 AAs    GLY47@G.s1h1.01 (  43) -    GLY52@G.s1h1.06 (48  ) (G.s1h1)
 G.H1   with      7 AAs    LYS53@G.H1.01   (  49) -    GLN59@G.H1.07   (55  ) (G.H1)
 H.HA   with     26 AAs    LYS88@H.HA.04   (  56) -   LEU113@H.HA.29   (81  ) (H.HA)
 H.hahb with      9 AAs   VAL114@H.hahb.01 (  82) -   PRO122@H.hahb.09 (90  ) (H.hahb)
 H.HB   with     14 AAs   GLU123@H.HB.01   (  91) -   ASN136@H.HB.14   (104 ) (H.HB)
 H.hbhc with      7 AAs   VAL137@H.hbhc.01 ( 105) -   PRO143@H.hbhc.15 (111 ) (H.hbhc)
 H.HC   with     12 AAs   PRO144@H.HC.01   ( 112) -   GLU155@H.HC.12   (123 ) (H.HC)
 H.hchd with      1 AAs   ASP156@H.hchd.01 ( 124) -   ASP156@H.hchd.01 (124 ) (H.hchd)
 H.HD   with     12 AAs   GLU157@H.HD.01   ( 125) -   GLU168@H.HD.12   (136 ) (H.HD)
 H.hdhe with      5 AAs   TYR169@H.hdhe.01 ( 137) -   ASP173@H.hdhe.05 (141 ) (H.hdhe)
 H.HE   with     13 AAs   CYS174@H.HE.01   ( 142) -   LYS186@H.HE.13   (154 ) (H.HE)
 H.hehf with      7 AAs   GLN187@H.hehf.01 ( 155) -   SER193@H.hehf.07 (161 ) (H.hehf)
 H.HF   with      6 AAs   ASP194@H.HF.01   ( 162) -   ARG199@H.HF.06   (167 ) (H.HF)
 G.hfs2 with      5 AAs   CYS200@G.hfs2.01 ( 168) -   GLY206@G.hfs2.07 (172 ) (G.hfs2) resSeq jumps
 G.S2   with      8 AAs   ILE207@G.S2.01   ( 173) -   VAL214@G.S2.08   (180 ) (G.S2)
 G.s2s3 with      2 AAs   ASP215@G.s2s3.01 ( 181) -   LYS216@G.s2s3.02 (182 ) (G.s2s3)
 G.S3   with      8 AAs   VAL217@G.S3.01   ( 183) -   VAL224@G.S3.08   (190 ) (G.S3)
 G.s3h2 with      3 AAs   GLY225@G.s3h2.01 ( 191) -   GLN227@G.s3h2.03 (193 ) (G.s3h2)
 G.H2   with     10 AAs   ARG228@G.H2.01   ( 194) -   CYS237@G.H2.10   (203 ) (G.H2)
 G.h2s4 with      5 AAs   PHE238@G.h2s4.01 ( 204) -   THR242@G.h2s4.05 (208 ) (G.h2s4)
 G.S4   with      7 AAs   ALA243@G.S4.01   ( 209) -   ALA249@G.S4.07   (215 ) (G.S4)
 G.s4h3 with      8 AAs   SER250@G.s4h3.01 ( 216) -   ASN264@G.s4h3.15 (223 ) (G.s4h3) resSeq jumps
 G.H3   with     18 AAs   ARG265@G.H3.01   ( 224) -   LEU282@G.H3.18   (241 ) (G.H3)
 G.h3s5 with      3 AAs   ARG283@G.h3s5.01 ( 242) -   ILE285@G.h3s5.03 (244 ) (G.h3s5)
 G.S5   with      7 AAs   SER286@G.S5.01   ( 245) -   ASN292@G.S5.07   (251 ) (G.S5)
 G.s5hg with      1 AAs   LYS293@G.s5hg.01 ( 252) -   LYS293@G.s5hg.01 (252 ) (G.s5hg)
 G.HG   with     17 AAs   GLN294@G.HG.01   ( 253) -   ASP310@G.HG.17   (269 ) (G.HG)
 G.hgh4 with     21 AAs   TYR311@G.hgh4.01 ( 270) -   ASP331@G.hgh4.21 (290 ) (G.hgh4)
 G.H4   with     16 AAs   PRO332@G.H4.01   ( 291) -   ARG347@G.H4.17   (306 ) (G.H4)
 G.h4s6 with     11 AAs   ILE348@G.h4s6.01 ( 307) -   TYR358@G.h4s6.20 (317 ) (G.h4s6)
 G.S6   with      5 AAs   CYS359@G.S6.01   ( 318) -   PHE363@G.S6.05   (322 ) (G.S6)
 G.s6h5 with      5 AAs   THR364@G.s6h5.01 ( 323) -   ASP368@G.s6h5.05 (327 ) (G.s6h5)
 G.H5   with     26 AAs   THR369@G.H5.01   ( 328) -   LEU394@G.H5.26   (353 ) (G.H5)
Select group 1: 0
Select group 2: 3

Will look for contacts in the interface between fragments
0
and
3.

Performing a first pass on the 100182 group_1-group_2 residue pairs to compute lower bounds on residue-residue distances via residue-COM distances.
Reduced to only 912 (from 100182) residue pairs for the computation of actual residue-residue distances:
```

```
The following 50 contacts capture 45.66 (~91%) of the total frequency 50.28 (over 107 contacts with nonzero frequency at 4.50 Angstrom).
As orientation value, the first 50 ctcs already capture 90.0% of 50.28.
The 50-th contact has a frequency of 0.52.

    freq              label                residues  fragments   sum
1   1.00  R385@G.H5.17   - K232@5.71x71   344 - 959    0 - 3    1.00
2   1.00  V217@G.S3.01   - F139@34.51x51  183 - 869    0 - 3    2.00
3   1.00  R385@G.H5.17   - Q229@5.68x68   344 - 956    0 - 3    3.00
4   1.00  D381@G.H5.13   - Q229@5.68x68   340 - 956    0 - 3    4.00
5   1.00  E392@G.H5.24   - T274@6.36x36   351 - 976    0 - 3    5.00
6   1.00  Y358@G.h4s6.20 - S236@5.75x75   317 - 963    0 - 3    6.00
7   1.00  D381@G.H5.13   - K232@5.71x71   340 - 959    0 - 3    7.00
8   1.00  Q384@G.H5.16   - I135@3.54x54   343 - 865    0 - 3    8.00
9   1.00  T350@G.h4s6.03 - R239@ICL3      309 - 966    0 - 3    9.00
10  1.00  L393@G.H5.25   - L275@6.37x37   352 - 977    0 - 3    9.99
11  1.00  Q384@G.H5.16   - Q229@5.68x68   343 - 956    0 - 3   10.99
12  0.99  H387@G.H5.19   - A134@3.53x53   346 - 864    0 - 3   11.98
13  0.99  L393@G.H5.25   - V222@5.61x61   352 - 949    0 - 3   12.98
14  0.99  L393@G.H5.25   - A271@6.33x33   352 - 973    0 - 3   13.97
15  0.99  L346@G.H4.16   - R239@ICL3      305 - 966    0 - 3   14.96
16  0.99  Y391@G.H5.23   - R131@3.50x50   350 - 861    0 - 3   15.95
17  0.99  L388@G.H5.20   - A226@5.65x65   347 - 953    0 - 3   16.93
18  0.99  L388@G.H5.20   - Q229@5.68x68   347 - 956    0 - 3   17.92
19  0.99  I383@G.H5.15   - P138@34.50x50  342 - 868    0 - 3   18.90
20  0.98  P138@H.hbhc.02 - Q142@34.54x54  106 - 872    0 - 3   19.89
21  0.98  H41@G.S1.02    - F139@34.51x51   37 - 869    0 - 3   20.87
22  0.98  Y358@G.h4s6.20 - I233@5.72x72   317 - 960    0 - 3   21.85
23  0.98  Q384@G.H5.16   - T136@3.55x55   343 - 866    0 - 3   22.82
24  0.97  Y360@G.S6.02   - S236@5.75x75   319 - 963    0 - 3   23.79
25  0.97  R385@G.H5.17   - I233@5.72x72   344 - 960    0 - 3   24.76
26  0.97  L394@G.H5.26   - L230@5.69x69   353 - 957    0 - 3   25.73
27  0.97  R380@G.H5.12   - F139@34.51x51  339 - 869    0 - 3   26.70
28  0.96  Y391@G.H5.23   - T274@6.36x36   350 - 976    0 - 3   27.66
29  0.96  Y391@G.H5.23   - I135@3.54x54   350 - 865    0 - 3   28.63
30  0.96  P138@H.hbhc.02 - S143@34.55x55  106 - 873    0 - 3   29.58
31  0.96  R38@G.hns1.02  - Q142@34.54x54   34 - 872    0 - 3   30.54
32  0.95  L388@G.H5.20   - I135@3.54x54   347 - 865    0 - 3   31.49
33  0.94  L393@G.H5.25   - T274@6.36x36   352 - 976    0 - 3   32.43
34  0.91  H387@G.H5.19   - P138@34.50x50  346 - 868    0 - 3   33.34
35  0.91  I383@G.H5.15   - F139@34.51x51  342 - 869    0 - 3   34.25
36  0.87  Q384@G.H5.16   - E225@5.64x64   343 - 952    0 - 3   35.12
37  0.86  F376@G.H5.08   - F139@34.51x51  335 - 869    0 - 3   35.99
38  0.86  D139@H.hbhc.03 - F139@34.51x51  107 - 869    0 - 3   36.85
39  0.86  S349@G.h4s6.02 - R239@ICL3      308 - 966    0 - 3   37.71
40  0.83  Q384@G.H5.16   - P138@34.50x50  343 - 868    0 - 3   38.54
41  0.82  E392@G.H5.24   - K270@6.32x32   351 - 972    0 - 3   39.36
42  0.81  H387@G.H5.19   - I135@3.54x54   346 - 865    0 - 3   40.17
43  0.80  L394@G.H5.26   - I233@5.72x72   353 - 960    0 - 3   40.97
44  0.79  Y358@G.h4s6.20 - E237@5.76x76   317 - 964    0 - 3   41.76
45  0.79  L393@G.H5.25   - I135@3.54x54   352 - 865    0 - 3   42.55
46  0.71  Y391@G.H5.23   - A134@3.53x53   350 - 864    0 - 3   43.26
47  0.65  D139@H.hbhc.03 - S143@34.55x55  107 - 873    0 - 3   43.91
48  0.65  E27@G.HN.44    - E62@12.48x48    23 - 792    0 - 3   44.56
49  0.58  P138@H.hbhc.02 - T146@4.38x38   106 - 876    0 - 3   45.14
50  0.52  L394@G.H5.26   - K270@6.32x32   353 - 972    0 - 3   45.66

             label  freq
1     L393@G.H5.25  4.71
2     Q384@G.H5.16  4.67
3     Y391@G.H5.23  3.62
4     R385@G.H5.17  2.97
5     L388@G.H5.20  2.92
6   Y358@G.h4s6.20  2.77
7     H387@G.H5.19  2.71
8   P138@H.hbhc.02  2.52
9     L394@G.H5.26  2.29
10    D381@G.H5.13  2.00
11    I383@G.H5.15  1.90
12    E392@G.H5.24  1.82
13  D139@H.hbhc.03  1.52
14    V217@G.S3.01  1.00
15  T350@G.h4s6.03  1.00
16    L346@G.H4.16  0.99
17     H41@G.S1.02  0.98
18    Y360@G.S6.02  0.97
19    R380@G.H5.12  0.97
20   R38@G.hns1.02  0.96
21    F376@G.H5.08  0.86
22  S349@G.h4s6.02  0.86
23     E27@G.HN.44  0.65

            label  freq
1   F139@34.51x51  5.59
2    I135@3.54x54  4.50
3    Q229@5.68x68  3.98
4    T274@6.36x36  2.90
5       R239@ICL3  2.85
6    I233@5.72x72  2.75
7   P138@34.50x50  2.73
8    K232@5.71x71  2.00
9    S236@5.75x75  1.97
10  Q142@34.54x54  1.94
11   A134@3.53x53  1.70
12  S143@34.55x55  1.61
13   K270@6.32x32  1.34
14   L275@6.37x37  1.00
15   V222@5.61x61  0.99
16   A271@6.33x33  0.99
17   R131@3.50x50  0.99
18   A226@5.65x65  0.99
19   T136@3.55x55  0.98
20   L230@5.69x69  0.97
21   E225@5.64x64  0.87
22   E237@5.76x76  0.79
23   E62@12.48x48  0.65
24   T146@4.38x38  0.58
```

Plot each residues's participation in the interface

In [6]:

```python
ifig = intf.plot_frequency_sums_as_bars(4.5, title_str = intf.name,
                                        list_by_interface=True,
                                        interface_vline=True);
ifig.figure.savefig("intf.svg")
```

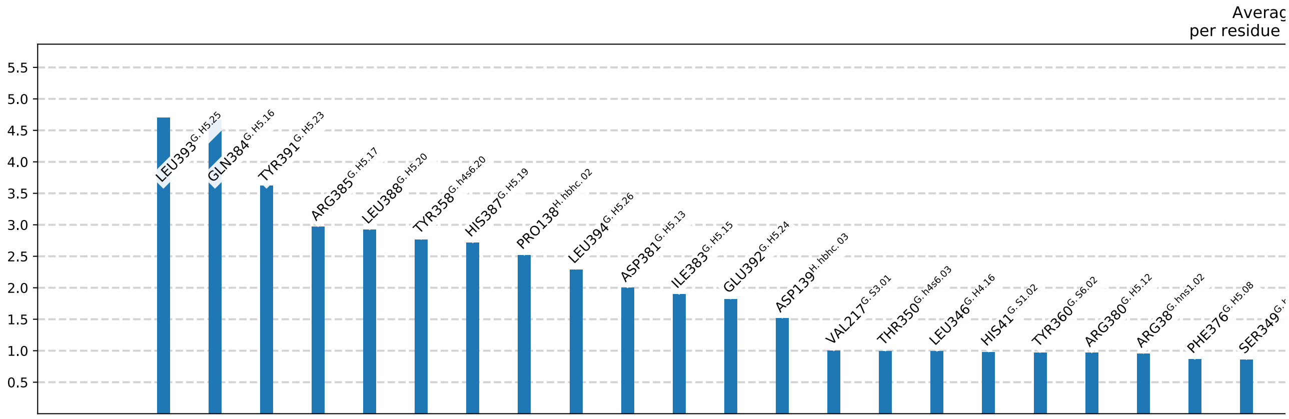

Plot contact matrix

In [7]:
```
ifig, iax = intf.plot_interface_frequency_matrix(4.5, grid=True, pixelsize=.5);
ifig.savefig("matrix.svg")
```

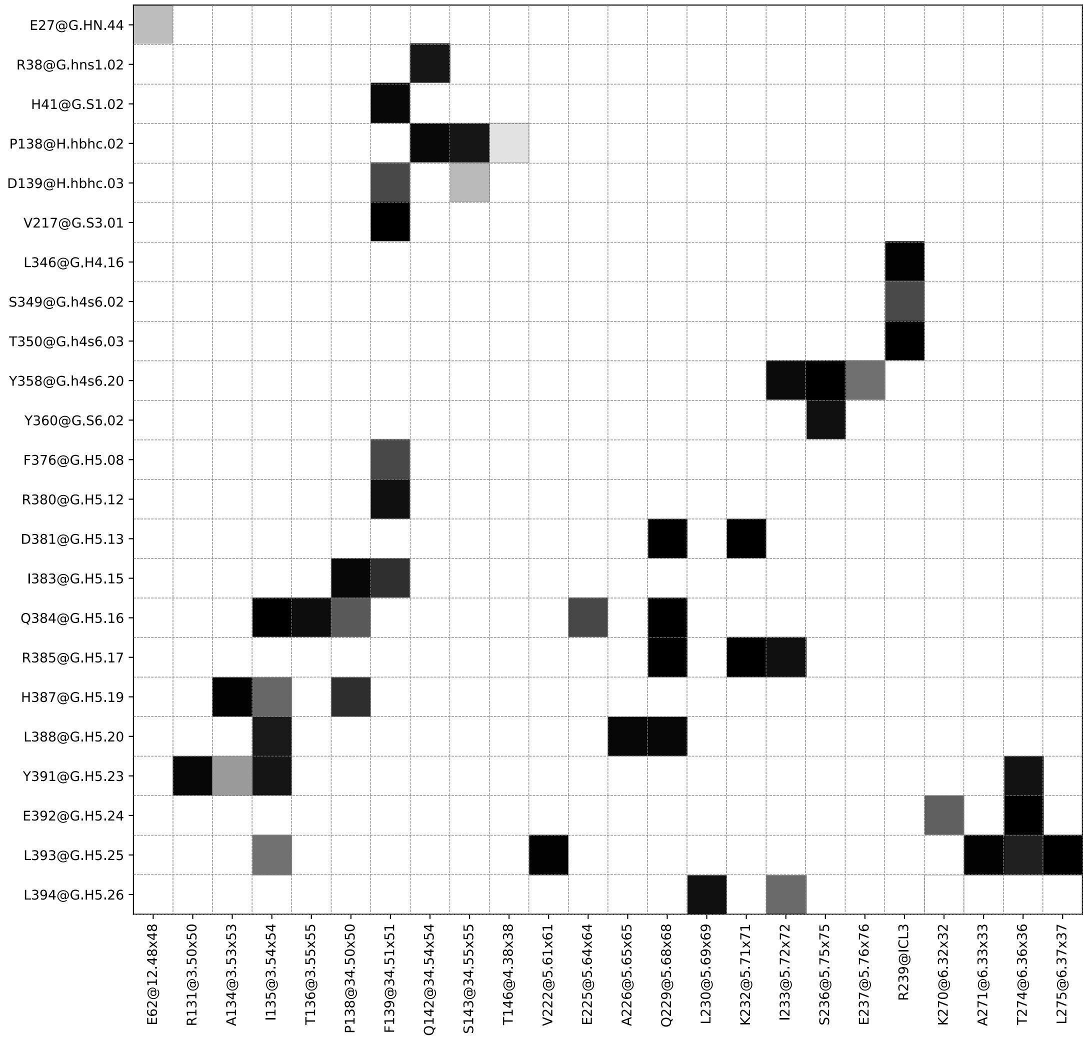

Flareplot

We combine a lot of information into one single flareplot:

- the molecular topology with sub-fragments and consensus labels,
- the secondary structure,
- the individual contact-pairs
- the participation of each residue in the interface.

In [8]:
```
ifig, iax, flareplot_attrs  = intf.plot_freqs_as_flareplot(4.5,
                                                            fragments=fragments, fragment_names = fragment_names,
                                                            scheme="consensus_sparse", consensus_maps=[GPCR, CGN],
                                                            aura=intf.frequency_sum_per_residue_idx_dict(4,return_array=True),
                                                            SS=True)
ifig.figure.savefig("flare.svg")
```

```
Drawing this many dots (270 residues + 18 padding spaces) in a panel 10.0 inches wide/high
forces too small dotsizes and fontsizes. If crowding effects occur, either reduce the
number of residues or increase the panel size
```

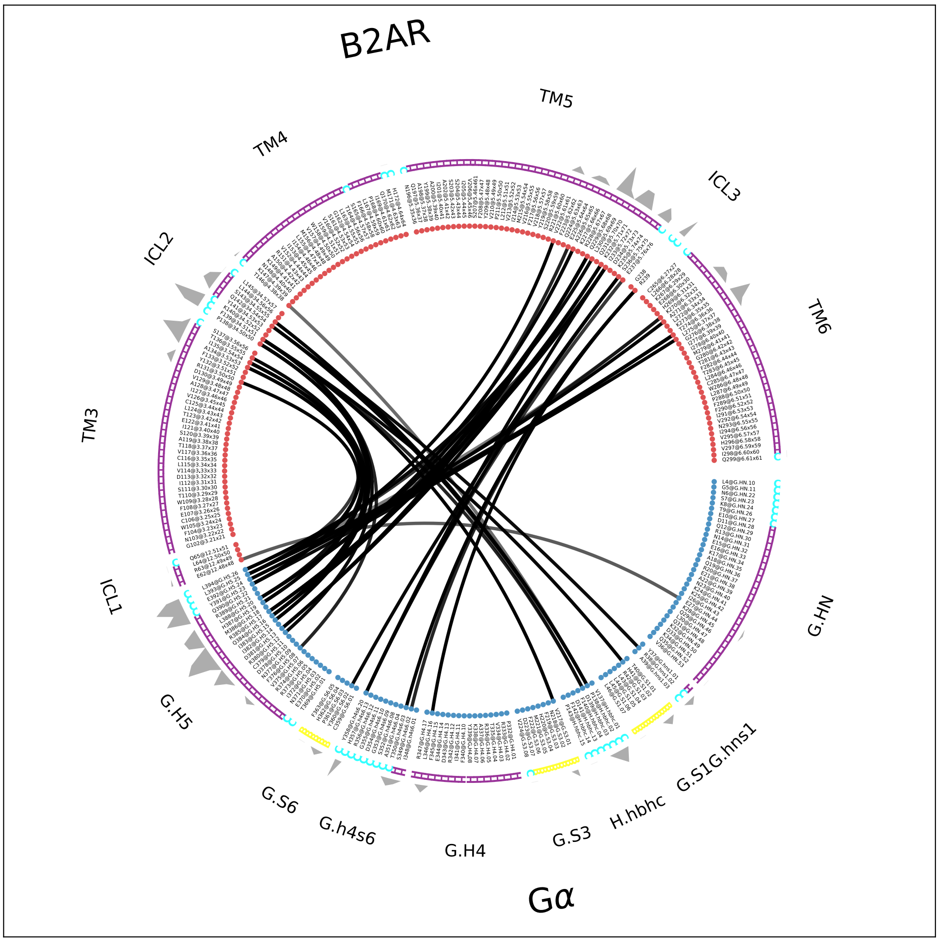

Coarse-Grained Frequencies and Flareplots

In [ ]:

In [9]:

```
ifig, iax, flareplot_attrs = intf.plot_freqs_as_flareplot(4.5,
                                                          fragments=fragments, fragment_names = fragment_names,
                                                          consensus_maps=[GPCR, CGN],
                                                          coarse_grain=True,
                                                         )
ifig.savefig("chord.svg",bbox_inches="tight")
freqs = intf.frequency_as_contact_matrix_CG(4.5, fragments=fragments, fragment_names = fragment_names,
                                            consensus_labelers=[GPCR, CGN],
                                            interface=True).round(1).replace(0,"")
freqs
```

Out[9]:

	ICL1	TM3	ICL2	TM4	TM5	ICL3	TM6
**G.HN**	0.6						
**G.hns1**			1.0				
**G.S1**			1.0				
**H.hbhc**			3.5	0.6			
**G.S3**			1.0				
**G.H4**						1.0	
**G.h4s6**					2.8	1.9	
**G.S6**					1.0		
**G.H5**		8.2	5.5		11.6		6.2

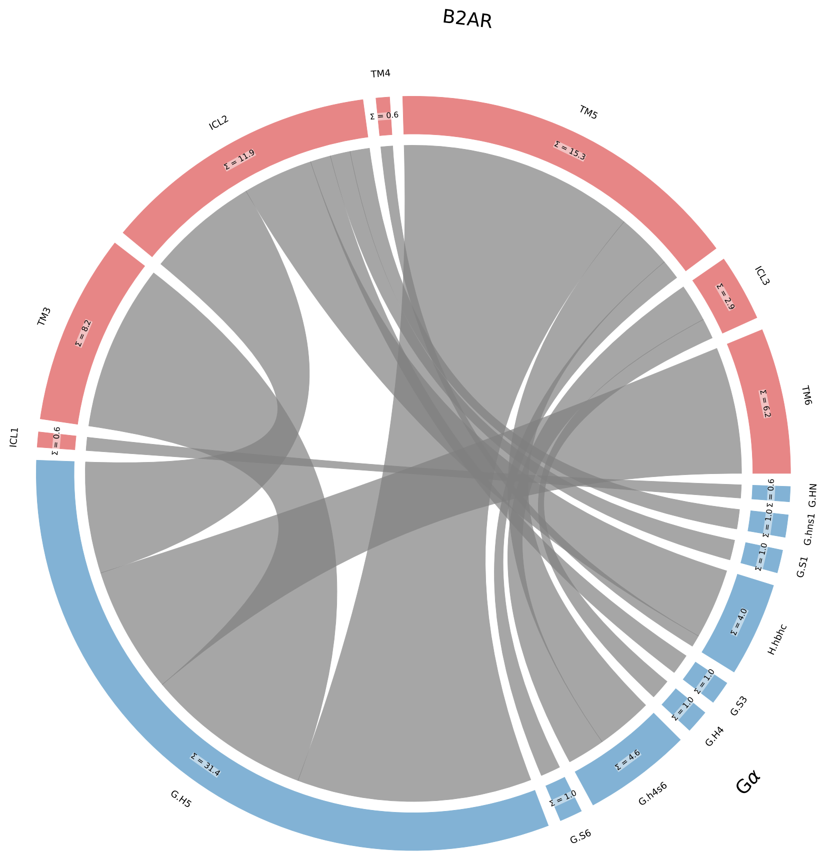

Grab a representative frame

This frame will be used to plot the interface frequencies as a 3D heatmap (see `frequency_to_bfactor` below).

In [10]:
```
repframe = intf.repframes(return_traj=True)[-1]
```

```
Returning frame 202 of traj nr. 0: <mdtraj.Trajectory with 280 frames, 8384 atoms, 1044 residues, and unitcells>
```

Save the interface as a heatmap and view externally

In [11]:
```
intf.frequency_to_bfactor(4.5, pdbfile="interface_heatmap.pdb",
                          geom=repframe,
                          interface_sign=True
                         );
```

```
Contact frequencies stored as signed bfactor in 'interface_heatmap.pdb'
```

Save all mdciao objects for later reuse

We can save all mdciao objects to numpy .npy (pickle) files and later reload them without having to compute everything again.

In [12]:
```
import numpy as np
np.save("GPCR.npy", GPCR)
np.save("CGN.npy",CGN)
np.save("intf.npy",intf)
```
