# Supplementary material for: mdciao: Accessible Analysis and Visualization of Molecular Dynamics Simulation Data
Source: PLoS Comput Biol. 2025 Apr 21;21(4):e1012837. doi: 10.1371/journal.pcbi.1012837 (PMC12011235; doi:10.1371/journal.pcbi.1012837)
Supplement: S2 Notebook — (PDF) [file pcbi.1012837.s008.pdf]

Binding-Pocket Interactions of Four EGFR Inhibitors

For this notebook, we use mdciao to visualize the binding-pocket interactions of four **Epidermal Growth Factor Receptor (EGFR) inhibitors**. EGFR is an important drug target with implications in cancer and inflammation (Wikipedia). It is a transmembrane protein with an extracellular receptor domain and an intracellular kinase domain.

The molecular dynamics (MD) data used here was generated by slightly modifying the notebook

- T019 · Molecular dynamics simulation

which is part of the impressive TeachOpenCADD collection, made available as teaching platform for computer-aided drug design by the Volkamer Lab at Saarland University, Saarbrücken.

The four inhibitors and structures are chosen from the following RCSB entries:

- *The crystal structure of EGFR T790M/C797S with the inhibitor HCD2892 (PDB ID 7VRE)*
- *EGFR kinase domain complexed with compound 20a (PDB ID 3W32)*
- *EGFR Kinase domain complexed with tak-285 (PDB ID 3POZ)*
- *Crystal Structure of EGFR(L858R/T790M/C797S) in complex with CH7233163 (PDB ID 6LUB)*

Please see the references at the bottom of the notebook for more information.

In [1]:
```
import mdciao
import os
import matplotlib
import nglview
from glob import glob
```

Consensus labeler object for KLIFS nomenclature

Since it will be used more than once, it is better to have it instantiated only once and reused many times. The only thing we need is the UniProt Accession Code of the EGFR, `P00533` .

In [2]:
```
KLIFS = mdciao.nomenclature.LabelerKLIFS("UniProtAC:P00533")
```

```
No local file ./KLIFS_UniProtAC:P00533.xlsx found, checking online in
https://klifs.net/api/kinase_ID?kinase_name=UniProtAC:P00533 ...https://klifs.net/api/structures_list?kinase_ID=406
done!
Please cite the following reference to the KLIF structural database:
 * Kanev et al, (2021) KLIFS: an overhaul after the first 5 years of supporting kinase research
   Nucleic Acids Research 49, D562--D569
   https://doi.org/10.1093/NAR/GKAA895
For more information, call mdciao.nomenclature.references()
/home/perezheg/miniconda3/lib/python3.11/site-packages/mdtraj/formats/pdb/pdbfile.py:206: UserWarning: Unlikely unit cell vectors detected in PDB file likely resul
ting from a dummy CRYST1 record. Discarding unit cell vectors.
  warnings.warn(
```

Download example data

In [3]:
```
if not os.path.exists("example_kinases"):
    mdciao.examples.fetch_example_data("EGFR");
```

```
Unzipping to 'example_kinases'
```

Guess molecular fragments

In [4]:
```
for pdb in sorted(glob("example_kinases/*.pdb")):
    print(pdb)
    mdciao.fragments.get_fragments(pdb)
    print()
```

```
example_kinases/topology.3POZ.pdb
Auto-detected fragments with method 'lig_resSeq+'
fragment      0 with    317 AAs    GLN701 (    0) -  LEU1017 (316 ) (0)
fragment      1 with      1 AAs      03P1 ( 317) -      03P1 (317 ) (1)

example_kinases/topology.3W32.pdb
Auto-detected fragments with method 'lig_resSeq+'
fragment      0 with    317 AAs    GLN701 (    0) -  LEU1017 (316 ) (0)
fragment      1 with      1 AAs      W321 ( 317) -      W321 (317 ) (1)

example_kinases/topology.6LUB.pdb
Auto-detected fragments with method 'lig_resSeq+'
fragment      0 with    323 AAs    GLY696 (    0) -  ILE1018 (322 ) (0)
fragment      1 with      1 AAs      EUX1 ( 323) -      EUX1 (323 ) (1)

example_kinases/topology.7VRE.pdb
Auto-detected fragments with method 'lig_resSeq+'
fragment      0 with    323 AAs    GLY696 (    0) -  ILE1018 (322 ) (0)
fragment      1 with      1 AAs      7VH1 ( 323) -      7VH1 (323 ) (1)
```

All three setups share the equivalent topology of kinase (fragment 0) and ligand (fragment 1):

- from PDB ID `3POZ` ligand `03P1`
- from PDB ID `3W32` ligand `W321`
- from PDB ID `6LUB` ligand `EUX1`
- from PDB ID `7VRE` ligand `7VH1`

For labelling purposes, create a mapping between PDB IDs and ligand names:

In [5]:
```
pdb2lig = {"3POZ" : "03P1",
           "3W32" : "W321",
           "6LUB" : "EUX1",
           "7VRE" : "7VH1"
          }
```

Compute the ligand-kinase interactions for the four inhibitors

In [6]:
```
binding_pocket = {}
for pdb in sorted(glob("example_kinases/*.pdb")):
    key = os.path.basename(pdb).split(".")[1]
```

```python
    key="%s@%s"%(pdb2lig[key], key)
    xtc = pdb.replace(".pdb",".xtc").replace("topology","trajectory")
    binding_pocket[key]=mdciao.cli.interface(xtc,
                                             pdb,
                                             fragment_names=["EGFR", "ligand"],
                                             KLIFS_string=KLIFS,
                                             ctc_control=1.0,
                                             interface_selection_1=[0],
                                             interface_selection_2=[1],
                                             accept_guess=True, figures=False, no_disk=True)
```

```
Will compute contact frequencies for trajectories:
example_kinases/trajectory.3POZ.xtc
 with a stride of 1 frames
Using method 'lig_resSeq+' these fragments were found
fragment EGFR   with    317 AAs   GLN701 (   0) -  LEU1017 (316 ) (EGFR)
fragment ligand with      1 AAs     03P1 ( 317) -     03P1 (317 ) (ligand)
The KLIFS-labels align best with fragments: [0] (first-last: GLN701-LEU1017).

Mapping the KLIFS fragments onto your topology:
 I      with      3 AAs   LYS716@I.1       (  15) -   LEU718@I.3       (17  ) (I)
 g.l    with      6 AAs   GLY719@g.l.4     (  18) -   GLY724@g.l.9     (23  ) (g.l)
 II     with      4 AAs   THR725@II.10     (  24) -   LYS728@II.13     (27  ) (II)
 III    with      6 AAs   VAL742@III.14    (  41) -   LEU747@III.19    (46  ) (III)
 αC     with     11 AAs   GLU758@αC.20     (  57) -   SER768@αC.30     (67  ) (αC)
 b.l    with      7 AAs   VAL769@b.l.31    (  68) -   ARG776@b.l.37    (75  ) (b.l) resSeq jumps
 IV     with      4 AAs   LEU777@IV.38     (  76) -   ILE780@IV.41     (79  ) (IV)
 V      with      3 AAs   GLN787@V.42      (  86) -   ILE789@V.44      (88  ) (V)
 GK     with      1 AAs   THR790@GK.45     (  89) -   THR790@GK.45     (89  ) (GK)
 hinge  with      3 AAs   GLN791@hinge.46  (  90) -   MET793@hinge.48  (92  ) (hinge)
 linker with      4 AAs   PRO794@linker.49 (  93) -   CYS797@linker.52 (96  ) (linker)
 αD     with      7 AAs   LEU798@αD.53     (  97) -   GLU804@αD.59     (103 ) (αD)
 αE     with      5 AAs   TYR827@αE.60     ( 126) -   ARG831@αE.64     (130 ) (αE)
 VI     with      3 AAs   ARG832@VI.65     ( 131) -   VAL834@VI.67     (133 ) (VI)
 c.l    with      8 AAs   HIS835@c.l.68    ( 134) -   ASN842@c.l.75    (141 ) (c.l)
 VII    with      3 AAs   VAL843@VII.76    ( 142) -   VAL845@VII.78    (144 ) (VII)
 VIII   with      1 AAs   ILE853@VIII.79   ( 152) -   ILE853@VIII.79   (152 ) (VIII)
 xDFG   with      4 AAs   THR854@xDFG.80   ( 153) -   GLY857@xDFG.83   (156 ) (xDFG)
 a.l    with      2 AAs   LEU858@a.l.84    ( 157) -   ALA859@a.l.85    (158 ) (a.l)
Select group 1: 0
Select group 2: 1

Will look for contacts in the interface between fragments
0
and
1.

Performing a first pass on the 317 group_1-group_2 residue pairs to compute lower bounds on residue-residue distances via residue-COM distances.
Reduced to only 220 (from 317) residue pairs for the computation of actual residue-residue distances:
```

```
The following 30 contacts capture 25.13 (~99%) of the total frequency 25.28 (over 36 contacts with nonzero frequency at 4.50 Angstrom).
As orientation value, the first 25 ctcs already capture 90.0% of 25.28.
The 25-th contact has a frequency of 0.55.

    freq             label              residues  fragments   sum
1   1.00  L792@hinge.47  - 03P1@ligand   91 - 317    0 - 1    1.00
2   1.00  M793@hinge.48  - 03P1@ligand   92 - 317    0 - 1    2.00
3   1.00  L777@IV.38     - 03P1@ligand   76 - 317    0 - 1    3.00
4   1.00  L844@VII.77    - 03P1@ligand  143 - 317    0 - 1    4.00
5   1.00  T854@xDFG.80   - 03P1@ligand  153 - 317    0 - 1    5.00
6   1.00  D855@xDFG.81   - 03P1@ligand  154 - 317    0 - 1    6.00
7   1.00  F856@xDFG.82   - 03P1@ligand  155 - 317    0 - 1    7.00
8   1.00  T790@GK.45     - 03P1@ligand   89 - 317    0 - 1    8.00
9   1.00  K745@III.17    - 03P1@ligand   44 - 317    0 - 1    9.00
10  1.00  C775@b.l.36    - 03P1@ligand   74 - 317    0 - 1   10.00
11  1.00  Q791@hinge.46  - 03P1@ligand   90 - 317    0 - 1   11.00
12  1.00  A743@III.15    - 03P1@ligand   42 - 317    0 - 1   12.00
13  1.00  L788@V.43      - 03P1@ligand   87 - 317    0 - 1   13.00
14  0.99  V726@II.11     - 03P1@ligand   25 - 317    0 - 1   13.99
15  0.99  R776@b.l.37    - 03P1@ligand   75 - 317    0 - 1   14.99
16  0.99  M766@αC.28     - 03P1@ligand   65 - 317    0 - 1   15.98
17  0.98  L718@I.3       - 03P1@ligand   17 - 317    0 - 1   16.95
18  0.91  I744@III.16    - 03P1@ligand   43 - 317    0 - 1   17.87
19  0.86  S720@g.l.5     - 03P1@ligand   19 - 317    0 - 1   18.72
20  0.85  R841@c.l.74    - 03P1@ligand  140 - 317    0 - 1   19.57
21  0.82  L858@a.l.84    - 03P1@ligand  157 - 317    0 - 1   20.39
22  0.81  G796@linker.51 - 03P1@ligand   95 - 317    0 - 1   21.20
23  0.80  F997@EGFR      - 03P1@ligand  296 - 317    0 - 1   22.00
24  0.68  I789@V.44      - 03P1@ligand   88 - 317    0 - 1   22.68
25  0.55  G719@g.l.4     - 03P1@ligand   18 - 317    0 - 1   23.22
26  0.50  G721@g.l.6     - 03P1@ligand   20 - 317    0 - 1   23.72
27  0.47  C797@linker.52 - 03P1@ligand   96 - 317    0 - 1   24.19
28  0.44  N842@c.l.75    - 03P1@ligand  141 - 317    0 - 1   24.63
29  0.36  I853@VIII.79   - 03P1@ligand  152 - 317    0 - 1   24.99
30  0.14  D800@αD.55     - 03P1@ligand   99 - 317    0 - 1   25.13

             label  freq
1    L792@hinge.47  1.00
2    M793@hinge.48  1.00
3       L777@IV.38  1.00
4      L844@VII.77  1.00
5     T854@xDFG.80  1.00
6     D855@xDFG.81  1.00
7     F856@xDFG.82  1.00
8       T790@GK.45  1.00
9      K745@III.17  1.00
10     C775@b.l.36  1.00
11   Q791@hinge.46  1.00
12     A743@III.15  1.00
13       L788@V.43  1.00
14      V726@II.11  0.99
15     R776@b.l.37  0.99
16      M766@αC.28  0.99
17        L718@I.3  0.98
18     I744@III.16  0.91
19      S720@g.l.5  0.86
20     R841@c.l.74  0.85
21     L858@a.l.84  0.82
22  G796@linker.51  0.81
23       F997@EGFR  0.80
24       I789@V.44  0.68
25      G719@g.l.4  0.55
26      G721@g.l.6  0.50
27  C797@linker.52  0.47
28     N842@c.l.75  0.44
29    I853@VIII.79  0.36
30      D800@αD.55  0.14

         label   freq
1  03P1@ligand  25.13

Will compute contact frequencies for trajectories:
example_kinases/trajectory.3W32.xtc
 with a stride of 1 frames
Using method 'lig_resSeq+' these fragments were found
fragment EGFR   with    317 AAs   GLN701 (   0) -  LEU1017 (316 ) (EGFR)
fragment ligand with      1 AAs     W321 ( 317) -     W321 (317 ) (ligand)
The KLIFS-labels align best with fragments: [0] (first-last: GLN701-LEU1017).

Mapping the KLIFS fragments onto your topology:
 I      with      3 AAs   LYS716@I.1       (  15) -   LEU718@I.3       (17  ) (I)
 g.l    with      6 AAs   GLY719@g.l.4     (  18) -   GLY724@g.l.9     (23  ) (g.l)
 II     with      4 AAs   THR725@II.10     (  24) -   LYS728@II.13     (27  ) (II)
 III    with      6 AAs   VAL742@III.14    (  41) -   LEU747@III.19    (46  ) (III)
 αC     with     11 AAs   GLU758@αC.20     (  57) -   SER768@αC.30     (67  ) (αC)
 b.l    with      7 AAs   VAL769@b.l.31    (  68) -   ARG776@b.l.37    (75  ) (b.l) resSeq jumps
 IV     with      4 AAs   LEU777@IV.38     (  76) -   ILE780@IV.41     (79  ) (IV)
 V      with      3 AAs   GLN787@V.42      (  86) -   ILE789@V.44      (88  ) (V)
 GK     with      1 AAs   THR790@GK.45     (  89) -   THR790@GK.45     (89  ) (GK)
 hinge  with      3 AAs   GLN791@hinge.46  (  90) -   MET793@hinge.48  (92  ) (hinge)
 linker with      4 AAs   PRO794@linker.49 (  93) -   CYS797@linker.52 (96  ) (linker)
 αD     with      7 AAs   LEU798@αD.53     (  97) -   GLU804@αD.59     (103 ) (αD)
 αE     with      5 AAs   TYR827@αE.60     ( 126) -   ARG831@αE.64     (130 ) (αE)
 VI     with      3 AAs   ARG832@VI.65     ( 131) -   VAL834@VI.67     (133 ) (VI)
 c.l    with      8 AAs   HIS835@c.l.68    ( 134) -   ASN842@c.l.75    (141 ) (c.l)
 VII    with      3 AAs   VAL843@VII.76    ( 142) -   VAL845@VII.78    (144 ) (VII)
 VIII   with      1 AAs   ILE853@VIII.79   ( 152) -   ILE853@VIII.79   (152 ) (VIII)
 xDFG   with      4 AAs   THR854@xDFG.80   ( 153) -   GLY857@xDFG.83   (156 ) (xDFG)
 a.l    with      2 AAs   LEU858@a.l.84    ( 157) -   ALA859@a.l.85    (158 ) (a.l)
Select group 1: 0
Select group 2: 1

Will look for contacts in the interface between fragments
0
and
1.

Performing a first pass on the 317 group_1-group_2 residue pairs to compute lower bounds on residue-residue distances via residue-COM distances.
Reduced to only 200 (from 317) residue pairs for the computation of actual residue-residue distances:
```

```
The following 31 contacts capture 26.59 (~100%) of the total frequency 26.67 (over 34 contacts with nonzero frequency at 4.50 Angstrom).
As orientation value, the first 25 ctcs already capture 90.0% of 26.67.
The 25-th contact has a frequency of 0.81.

    freq            label              residues  fragments   sum
1   1.00  T790@GK.45     - W321@ligand   89 - 317    0 - 1    1.00
2   1.00  L792@hinge.47  - W321@ligand   91 - 317    0 - 1    2.00
3   1.00  C775@b.l.36    - W321@ligand   74 - 317    0 - 1    3.00
4   1.00  L788@V.43      - W321@ligand   87 - 317    0 - 1    4.00
5   1.00  T854@xDFG.80   - W321@ligand  153 - 317    0 - 1    5.00
6   1.00  D855@xDFG.81   - W321@ligand  154 - 317    0 - 1    6.00
7   1.00  F856@xDFG.82   - W321@ligand  155 - 317    0 - 1    7.00
8   1.00  K745@III.17    - W321@ligand   44 - 317    0 - 1    8.00
9   1.00  L777@IV.38     - W321@ligand   76 - 317    0 - 1    9.00
10  1.00  Q791@hinge.46  - W321@ligand   90 - 317    0 - 1   10.00
11  1.00  A743@III.15    - W321@ligand   42 - 317    0 - 1   11.00
12  1.00  M793@hinge.48  - W321@ligand   92 - 317    0 - 1   12.00
13  1.00  V726@II.11     - W321@ligand   25 - 317    0 - 1   13.00
14  1.00  R776@b.l.37    - W321@ligand   75 - 317    0 - 1   14.00
15  1.00  M766@αC.28     - W321@ligand   65 - 317    0 - 1   15.00
16  1.00  L844@VII.77    - W321@ligand  143 - 317    0 - 1   16.00
17  1.00  G719@g.l.4     - W321@ligand   18 - 317    0 - 1   16.99
18  0.99  L718@I.3       - W321@ligand   17 - 317    0 - 1   17.99
19  0.98  G796@linker.51 - W321@ligand   95 - 317    0 - 1   18.97
20  0.97  S720@g.l.5     - W321@ligand   19 - 317    0 - 1   19.94
21  0.88  I744@III.16    - W321@ligand   43 - 317    0 - 1   20.82
22  0.86  I789@V.44      - W321@ligand   88 - 317    0 - 1   21.68
23  0.83  L858@a.l.84    - W321@ligand  157 - 317    0 - 1   22.51
24  0.82  C797@linker.52 - W321@ligand   96 - 317    0 - 1   23.33
25  0.81  L1001@EGFR     - W321@ligand  300 - 317    0 - 1   24.14
26  0.74  G721@g.l.6     - W321@ligand   20 - 317    0 - 1   24.89
27  0.54  I853@VIII.79   - W321@ligand  152 - 317    0 - 1   25.42
28  0.47  R841@c.l.74    - W321@ligand  140 - 317    0 - 1   25.90
29  0.24  F997@EGFR      - W321@ligand  296 - 317    0 - 1   26.14
30  0.23  D800@αD.55     - W321@ligand   99 - 317    0 - 1   26.38
31  0.22  G724@g.l.9     - W321@ligand   23 - 317    0 - 1   26.59

             label  freq
1       T790@GK.45  1.00
2    L792@hinge.47  1.00
3      C775@b.l.36  1.00
4        L788@V.43  1.00
5     T854@xDFG.80  1.00
6     D855@xDFG.81  1.00
7     F856@xDFG.82  1.00
8      K745@III.17  1.00
9       L777@IV.38  1.00
10   Q791@hinge.46  1.00
11     A743@III.15  1.00
12   M793@hinge.48  1.00
13      V726@II.11  1.00
14     R776@b.l.37  1.00
15      M766@αC.28  1.00
16     L844@VII.77  1.00
17      G719@g.l.4  1.00
18        L718@I.3  0.99
19  G796@linker.51  0.98
20      S720@g.l.5  0.97
21     I744@III.16  0.88
22       I789@V.44  0.86
23     L858@a.l.84  0.83
24  C797@linker.52  0.82
25      L1001@EGFR  0.81
26      G721@g.l.6  0.74
27    I853@VIII.79  0.54
28     R841@c.l.74  0.47
29       F997@EGFR  0.24
30      D800@αD.55  0.23
31      G724@g.l.9  0.22

         label   freq
1  W321@ligand  26.59

Will compute contact frequencies for trajectories:
example_kinases/trajectory.6LUB.xtc
 with a stride of 1 frames
Using method 'lig_resSeq+' these fragments were found
fragment EGFR   with    323 AAs   GLY696 (   0) -  ILE1018 (322 ) (EGFR)
fragment ligand with      1 AAs     EUX1 ( 323) -     EUX1 (323 ) (ligand)
The KLIFS-labels align best with fragments: [0] (first-last: GLY696-ILE1018).

Mapping the KLIFS fragments onto your topology:
 I      with      3 AAs   LYS716@I.1       (  20) -   LEU718@I.3       (22  ) (I)
 g.l    with      6 AAs   GLY719@g.l.4     (  23) -   GLY724@g.l.9     (28  ) (g.l)
 II     with      4 AAs   THR725@II.10     (  29) -   LYS728@II.13     (32  ) (II)
 III    with      6 AAs   VAL742@III.14    (  46) -   LEU747@III.19    (51  ) (III)
 αC     with     11 AAs   GLU758@αC.20     (  62) -   SER768@αC.30     (72  ) (αC)
 b.l    with      7 AAs   VAL769@b.l.31    (  73) -   ARG776@b.l.37    (80  ) (b.l) resSeq jumps
 IV     with      4 AAs   LEU777@IV.38     (  81) -   ILE780@IV.41     (84  ) (IV)
 V      with      3 AAs   GLN787@V.42      (  91) -   ILE789@V.44      (93  ) (V)
 GK     with      1 AAs   MET790@GK.45     (  94) -   MET790@GK.45     (94  ) (GK)
 hinge  with      3 AAs   GLN791@hinge.46  (  95) -   MET793@hinge.48  (97  ) (hinge)
 linker with      4 AAs   PRO794@linker.49 (  98) -   SER797@linker.52 (101 ) (linker)
 αD     with      7 AAs   LEU798@αD.53     ( 102) -   GLU804@αD.59     (108 ) (αD)
 αE     with      5 AAs   TYR827@αE.60     ( 131) -   ARG831@αE.64     (135 ) (αE)
 VI     with      3 AAs   ARG832@VI.65     ( 136) -   VAL834@VI.67     (138 ) (VI)
 c.l    with      8 AAs   HIS835@c.l.68    ( 139) -   ASN842@c.l.75    (146 ) (c.l)
 VII    with      3 AAs   VAL843@VII.76    ( 147) -   VAL845@VII.78    (149 ) (VII)
 VIII   with      1 AAs   ILE853@VIII.79   ( 157) -   ILE853@VIII.79   (157 ) (VIII)
 xDFG   with      4 AAs   THR854@xDFG.80   ( 158) -   GLY857@xDFG.83   (161 ) (xDFG)
 a.l    with      2 AAs   ARG858@a.l.84    ( 162) -   ALA859@a.l.85    (163 ) (a.l)
Select group 1: 0
Select group 2: 1

Will look for contacts in the interface between fragments
0
and
1.

Performing a first pass on the 323 group_1-group_2 residue pairs to compute lower bounds on residue-residue distances via residue-COM distances.
Reduced to only 190 (from 323) residue pairs for the computation of actual residue-residue distances:
```

```
The following 25 contacts capture 20.50 (~98%) of the total frequency 20.83 (over 39 contacts with nonzero frequency at 4.50 Angstrom).
As orientation value, the first 20 ctcs already capture 90.0% of 20.83.
The 20-th contact has a frequency of 0.64.

    freq             label              residues  fragments   sum
1   1.00  G796@linker.51 - EUX1@ligand  100 - 323    0 - 1    1.00
2   1.00  V726@II.11     - EUX1@ligand   30 - 323    0 - 1    2.00
3   1.00  P794@linker.49 - EUX1@ligand   98 - 323    0 - 1    3.00
4   1.00  L718@I.3       - EUX1@ligand   22 - 323    0 - 1    4.00
5   1.00  M793@hinge.48  - EUX1@ligand   97 - 323    0 - 1    5.00
6   1.00  L844@VII.77    - EUX1@ligand  148 - 323    0 - 1    6.00
7   1.00  L792@hinge.47  - EUX1@ligand   96 - 323    0 - 1    7.00
8   1.00  Q791@hinge.46  - EUX1@ligand   95 - 323    0 - 1    8.00
9   1.00  M790@GK.45     - EUX1@ligand   94 - 323    0 - 1    9.00
10  1.00  A743@III.15    - EUX1@ligand   47 - 323    0 - 1   10.00
11  0.98  G724@g.l.9     - EUX1@ligand   28 - 323    0 - 1   10.97
12  0.97  T854@xDFG.80   - EUX1@ligand  158 - 323    0 - 1   11.94
13  0.96  K745@III.17    - EUX1@ligand   49 - 323    0 - 1   12.90
14  0.95  S797@linker.52 - EUX1@ligand  101 - 323    0 - 1   13.85
15  0.92  G721@g.l.6     - EUX1@ligand   25 - 323    0 - 1   14.78
16  0.92  K728@II.13     - EUX1@ligand   32 - 323    0 - 1   15.70
17  0.92  G719@g.l.4     - EUX1@ligand   23 - 323    0 - 1   16.61
18  0.88  T725@II.10     - EUX1@ligand   29 - 323    0 - 1   17.49
19  0.82  C775@b.l.36    - EUX1@ligand   79 - 323    0 - 1   18.31
20  0.64  F795@linker.50 - EUX1@ligand   99 - 323    0 - 1   18.95
21  0.48  D855@xDFG.81   - EUX1@ligand  159 - 323    0 - 1   19.43
22  0.43  S720@g.l.5     - EUX1@ligand   24 - 323    0 - 1   19.86
23  0.32  D800@αD.55     - EUX1@ligand  104 - 323    0 - 1   20.17
24  0.17  R841@c.l.74    - EUX1@ligand  145 - 323    0 - 1   20.34
25  0.15  L1001@EGFR     - EUX1@ligand  305 - 323    0 - 1   20.50

             label  freq
1   G796@linker.51  1.00
2       V726@II.11  1.00
3   P794@linker.49  1.00
4         L718@I.3  1.00
5    M793@hinge.48  1.00
6      L844@VII.77  1.00
7    L792@hinge.47  1.00
8    Q791@hinge.46  1.00
9       M790@GK.45  1.00
10     A743@III.15  1.00
11      G724@g.l.9  0.98
12    T854@xDFG.80  0.97
13     K745@III.17  0.96
14  S797@linker.52  0.95
15      G721@g.l.6  0.92
16      K728@II.13  0.92
17      G719@g.l.4  0.92
18      T725@II.10  0.88
19     C775@b.l.36  0.82
20  F795@linker.50  0.64
21    D855@xDFG.81  0.48
22      S720@g.l.5  0.43
23      D800@αD.55  0.32
24     R841@c.l.74  0.17
25      L1001@EGFR  0.15

         label  freq
1  EUX1@ligand  20.5

Will compute contact frequencies for trajectories:
example_kinases/trajectory.7VRE.xtc
 with a stride of 1 frames
Using method 'lig_resSeq+' these fragments were found
fragment EGFR   with    323 AAs   GLY696 (   0) -  ILE1018 (322 ) (EGFR)
fragment ligand with      1 AAs     7VH1 ( 323) -     7VH1 (323 ) (ligand)
The KLIFS-labels align best with fragments: [0] (first-last: GLY696-ILE1018).

Mapping the KLIFS fragments onto your topology:
 I      with      3 AAs   LYS716@I.1       (  20) -   LEU718@I.3       (22  ) (I)
 g.l    with      6 AAs   GLY719@g.l.4     (  23) -   GLY724@g.l.9     (28  ) (g.l)
 II     with      4 AAs   THR725@II.10     (  29) -   LYS728@II.13     (32  ) (II)
 III    with      6 AAs   VAL742@III.14    (  46) -   LEU747@III.19    (51  ) (III)
 αC     with     11 AAs   GLU758@αC.20     (  62) -   SER768@αC.30     (72  ) (αC)
 b.l    with      7 AAs   VAL769@b.l.31    (  73) -   ARG776@b.l.37    (80  ) (b.l) resSeq jumps
 IV     with      4 AAs   LEU777@IV.38     (  81) -   ILE780@IV.41     (84  ) (IV)
 V      with      3 AAs   GLN787@V.42      (  91) -   ILE789@V.44      (93  ) (V)
 GK     with      1 AAs   MET790@GK.45     (  94) -   MET790@GK.45     (94  ) (GK)
 hinge  with      3 AAs   GLN791@hinge.46  (  95) -   MET793@hinge.48  (97  ) (hinge)
 linker with      4 AAs   PRO794@linker.49 (  98) -   SER797@linker.52 (101 ) (linker)
 αD     with      7 AAs   LEU798@αD.53     ( 102) -   GLU804@αD.59     (108 ) (αD)
 αE     with      5 AAs   TYR827@αE.60     ( 131) -   ARG831@αE.64     (135 ) (αE)
 VI     with      3 AAs   ARG832@VI.65     ( 136) -   VAL834@VI.67     (138 ) (VI)
 c.l    with      8 AAs   HIS835@c.l.68    ( 139) -   ASN842@c.l.75    (146 ) (c.l)
 VII    with      3 AAs   VAL843@VII.76    ( 147) -   VAL845@VII.78    (149 ) (VII)
 VIII   with      1 AAs   ILE853@VIII.79   ( 157) -   ILE853@VIII.79   (157 ) (VIII)
 xDFG   with      4 AAs   THR854@xDFG.80   ( 158) -   GLY857@xDFG.83   (161 ) (xDFG)
 a.l    with      2 AAs   LEU858@a.l.84    ( 162) -   ALA859@a.l.85    (163 ) (a.l)
Select group 1: 0
Select group 2: 1

Will look for contacts in the interface between fragments
0
and
1.

Performing a first pass on the 323 group_1-group_2 residue pairs to compute lower bounds on residue-residue distances via residue-COM distances.
Reduced to only 212 (from 323) residue pairs for the computation of actual residue-residue distances:
```

```
The following 23 contacts capture 16.70 (~99%) of the total frequency 16.79 (over 30 contacts with nonzero frequency at 4.50 Angstrom).
As orientation value, the first 17 ctcs already capture 90.0% of 16.79.
The 17-th contact has a frequency of 0.53.

    freq             label              residues  fragments   sum
1   1.00  L718@I.3       - 7VH1@ligand   22 - 323    0 - 1    1.00
2   1.00  M793@hinge.48  - 7VH1@ligand   97 - 323    0 - 1    2.00
3   1.00  L792@hinge.47  - 7VH1@ligand   96 - 323    0 - 1    3.00
4   1.00  V726@II.11     - 7VH1@ligand   30 - 323    0 - 1    4.00
5   1.00  A743@III.15    - 7VH1@ligand   47 - 323    0 - 1    5.00
6   1.00  P794@linker.49 - 7VH1@ligand   98 - 323    0 - 1    6.00
7   1.00  G796@linker.51 - 7VH1@ligand  100 - 323    0 - 1    6.99
8   1.00  Q791@hinge.46  - 7VH1@ligand   95 - 323    0 - 1    7.99
9   0.99  L844@VII.77    - 7VH1@ligand  148 - 323    0 - 1    8.98
10  0.96  F723@g.l.8     - 7VH1@ligand   27 - 323    0 - 1    9.93
11  0.93  G719@g.l.4     - 7VH1@ligand   23 - 323    0 - 1   10.86
12  0.90  M790@GK.45     - 7VH1@ligand   94 - 323    0 - 1   11.76
13  0.88  T854@xDFG.80   - 7VH1@ligand  158 - 323    0 - 1   12.64
14  0.79  D855@xDFG.81   - 7VH1@ligand  159 - 323    0 - 1   13.44
15  0.76  K745@III.17    - 7VH1@ligand   49 - 323    0 - 1   14.20
16  0.68  L1001@EGFR     - 7VH1@ligand  305 - 323    0 - 1   14.88
17  0.53  R841@c.l.74    - 7VH1@ligand  145 - 323    0 - 1   15.41
18  0.44  F795@linker.50 - 7VH1@ligand   99 - 323    0 - 1   15.85
19  0.20  C775@b.l.36    - 7VH1@ligand   79 - 323    0 - 1   16.05
20  0.19  N842@c.l.75    - 7VH1@ligand  146 - 323    0 - 1   16.24
21  0.18  K728@II.13     - 7VH1@ligand   32 - 323    0 - 1   16.41
22  0.16  S797@linker.52 - 7VH1@ligand  101 - 323    0 - 1   16.58
23  0.12  D800@αD.55     - 7VH1@ligand  104 - 323    0 - 1   16.70

             label  freq
1         L718@I.3  1.00
2    M793@hinge.48  1.00
3    L792@hinge.47  1.00
4       V726@II.11  1.00
5      A743@III.15  1.00
6   P794@linker.49  1.00
7   G796@linker.51  1.00
8    Q791@hinge.46  1.00
9      L844@VII.77  0.99
10      F723@g.l.8  0.96
11      G719@g.l.4  0.93
12      M790@GK.45  0.90
13    T854@xDFG.80  0.88
14    D855@xDFG.81  0.79
15     K745@III.17  0.76
16      L1001@EGFR  0.68
17     R841@c.l.74  0.53
18  F795@linker.50  0.44
19     C775@b.l.36  0.20
20     N842@c.l.75  0.19
21      K728@II.13  0.18
22  S797@linker.52  0.16
23      D800@αD.55  0.12

         label  freq
1  7VH1@ligand  16.7
```

Compare interactions across the four compounds in a violinplot

Additionally, we will display *representative* geometries directly on the violinplots via their residue-residue distance-values. Subsequently, we will view these geometries in 3D

In [7]:
```python
colors = mdciao.plots.color_dict_guesser("tab10", binding_pocket.keys())
myfig, myax, keys, representatives = mdciao.plots.compare_violins(binding_pocket,
                                                                  colors=colors,
                                                                  anchor="ligand",
                                                                  ctc_cutoff_Ang=4.5,
                                                                  mutations_dict={
                                                                      "EUX1": "ligand",
                                                                      "7VH1": "ligand",
                                                                      "W321": "ligand",
                                                                      "03P1": "ligand"
                                                                  },
                                                                  defrag=None,
                                                                  sort_by="residue",
                                                                  inch_per_contacts=.80,
                                                                  legend_rows=2,
                                                                  representatives=True,
                                                                  figsize=(11.5,3.5)
                                                         )
myax.set_title("binding pocket interactions"
               "\nfor 4 different EGFR inhibitors")
myfig.tight_layout()
#myfig.savefig("EGFR.png", bbox_inches="tight")
```

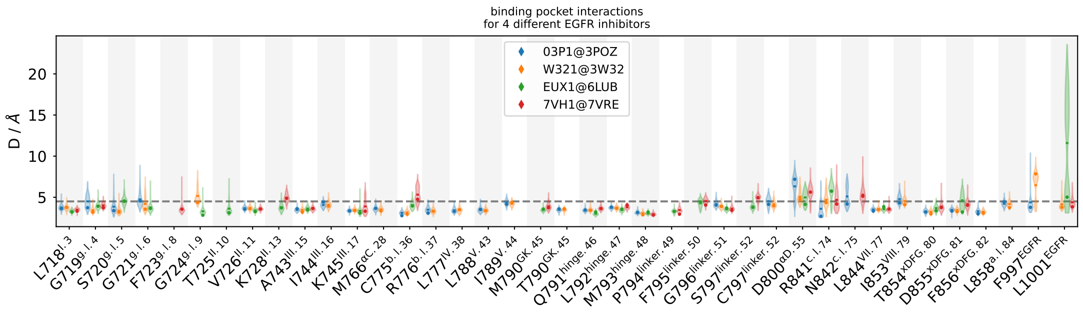

Show the representative geometries

The object `representatives` is a dictionary contaning the geometries behind the small dots inside the violins of the previous figure, using the repframes method. In the next cells we will first align them and then overlap them using the KLIFS nomenclature.

Superpose structures using the KLIFs alignment labels

This way, the alignment will be particularly good in the binding pocket

In [8]:
```
KLIFS_alignment = mdciao.nomenclature.AlignerConsensus({key : KLIFS for key in binding_pocket.keys()},
                                                        tops={key : bp.top for key, bp in binding_pocket.items()})

KLIFS_alignment.AAresSeq
```

Out[8]:

	consensus	03P1@3POZ	W321@3W32	EUX1@6LUB	7VH1@7VRE
**0**	I.1	K716	K716	K716	K716
**1**	I.2	V717	V717	V717	V717
**2**	I.3	L718	L718	L718	L718
**3**	g.l.4	G719	G719	G719	G719
**4**	g.l.5	S720	S720	S720	S720
**...**	...	...	...	...	...
**80**	xDFG.81	D855	D855	D855	D855
**81**	xDFG.82	F856	F856	F856	F856
**82**	xDFG.83	G857	G857	G857	G857
**83**	a.l.84	L858	L858	R858	L858
**84**	a.l.85	A859	A859	A859	A859

85 rows × 5 columns

In [9]:
```
# We can directly get CA indices to map atoms
KLIFS_alignment.CAidxs
```

Out[9]:

	consensus	03P1@3POZ	W321@3W32	EUX1@6LUB	7VH1@7VRE
**0**	I.1	280	280	340	340
**1**	I.2	302	302	362	362
**2**	I.3	318	318	378	378
**3**	g.l.4	337	337	397	397
**4**	g.l.5	344	344	404	404
**...**	...	...	...	...	...
**80**	xDFG.81	2515	2515	2578	2578
**81**	xDFG.82	2527	2527	2590	2590
**82**	xDFG.83	2547	2547	2610	2610
**83**	a.l.84	2554	2554	2617	2617
**84**	a.l.85	2573	2573	2641	2636

85 rows × 5 columns

In [10]:
```
ref_key = "W321@3W32" # We take this one but could be any one
ref_geom = representatives[ref_key]
for key, geom in representatives.items():
    if key!=ref_key:
        ref_CAs, key_CAs = KLIFS_alignment.CAidxs[[ref_key, key]].values.T.astype(int)
        geom.superpose(ref_geom, atom_indices=key_CAs, ref_atom_indices=ref_CAs)
```

Visualize residues with different behaviors in each compound

For example, residues

- `775@b.l.36`
- `841@c.l.74`
- `855@xDFG.81`
- `997@EGFR` (doesn't have a KLIFS label)

In [12]:
```
colors = {key: matplotlib.colors.to_hex(col) for key, col in colors.items()}
iwd = nglview.NGLWidget()
for ii, (key, rep) in enumerate(representatives.items()):
    iwd.add_trajectory(rep)
    iwd.clear_representations(component=ii)
    iwd.add_cartoon(color="white", component=ii)
    iwd.add_licorice(color=colors[key], component=ii, selection="(775 841 855 997) and not Hydrogen", radius=.1)
    iwd.add_ball_and_stick(color=colors[key], component=ii,
                          selection="not protein and not Hydrogen",
                           radius=.1,
                          )
iwd
```

References

- The crystal structure of EGFR T790M/C797S with the inhibitor HCD2892 (PDB ID 7VRE)
  - Chen, H., Lai, M., Zhang, T., Chen, Y., Tong, L., Zhu, S., ... Ding, K. (2022).
    Conformational Constrained 4-(1-Sulfonyl-3-indol)yl-2-phenylaminopyrimidine Derivatives as New Fourth-Generation Epidermal Growth Factor Receptor Inhibitors Targeting T790M/C797S Mutations.
    Journal of Medicinal Chemistry, 65(9), 6840–6858.
    https://doi.org/10.1021/acs.jmedchem.2c00168
- EGFR kinase domain complexed with compound 20a (PDB ID 3W32)
  - Kawakita, Y., Seto, M., Ohashi, T., Tamura, T., Yusa, T., Miki, H., ... Ishikawa, T. (2013).
    Design and synthesis of novel pyrimido[4,5- b]azepine derivatives as HER2/EGFR dual inhibitors.
    Bioorganic & Medicinal Chemistry, 21(8), 2250–2261.
    https://doi.org/10.1016/j.bmc.2013.02.014
- EGFR Kinase domain complexed with tak-285 (PDB ID 3POZ)
  - Aertgeerts, K., Skene, R., Yano, J., Sang, B. C., Zou, H., Snell, G., ... Sogabe, S. (2011).
    Structural analysis of the mechanism of inhibition and allosteric activation of the kinase domain of HER2 protein.

Journal of Biological Chemistry, 286(21), 18756–18765.
https://doi.org/10.1074/jbc.M110.206193

- Crystal Structure of EGFR(L858R/T790M/C797S) in complex with CH7233163 (PDB ID 6LUB)
  - Kashima, K., Kawauchi, H., Tanimura, H., Tachibana, Y., Chiba, T., Torizawa, T., & Sakamoto, H. (2020).
    CH7233163 Overcomes Osimertinib-Resistant EGFR-Del19/T790M/C797S Mutation.
    Molecular Cancer Therapeutics, 19(11), 2288–2297.
    https://doi.org/10.1158/1535-7163.MCT-20-0229
