# Supplementary material for: mdciao: Accessible Analysis and Visualization of Molecular Dynamics Simulation Data
Source: PLoS Comput Biol. 2025 Apr 21;21(4):e1012837. doi: 10.1371/journal.pcbi.1012837 (PMC12011235; doi:10.1371/journal.pcbi.1012837)
Supplement: Attachment — Submitted filename: point_by_point_response.pdf [file pcbi.1012837.s011.pdf]

REVIEWER 1

Review of "mdciao: An Open-Source Tool for Molecular Dynamics Simulation Analysis"

The paper presents "mdciao," an open-source command line tool and Python API designed for the analysis and representation of molecular dynamics (MD) simulation data. While the tool offers a user-friendly interface and a range of pre-packaged solutions for both non-expert and expert users, several critical areas require improvement to enhance the paper's clarity and comprehensiveness.

We thank the reviewer for their work and were happy to hear that they find mdciao a promising tool for MD simulations. As can be seen below, we have taken their suggestion to heart and now detail the mathematical expressions underpinning the frequency computation, central to mdciao. As to the other issues raised by this referee, they all point almost all in the same direction, which has no issue with the correctness or appropriateness of mdciao functionalities, but rather the scope and context in which they are presented.

Comparison with Existing Tools: One significant omission in the paper is a comparison of "mdciao" with other existing tools in the field. A detailed comparison would provide readers with a clearer understanding of the unique advantages and potential limitations of "mdciao" relative to other available software. This comparison should include performance metrics, type of use, and the range of functionalities offered.

We thank the reviewer for this comment, and have provided a table with performance numbers over a sample of MD datasets in the new Suppl. Table 2. Furthermore, while we can't hardly object to benchmarks, reviews, comparisons and detailed explanations producing useful material for lookup and reference, we kindly disagree with their inclusion in the manuscript, believing that they are squarely outside its intended scope and contrary to its brief, concise nature. The value proposition of mdciao is not "to do what other's cant", but simplify as much as possible "what others offer". Hence, a feature comparison would be unfair to the modules providing the very functionalities that mdciao relies on.

We respectfully posit that the "price" to present a software package cannot be to produce a benchmarking or a review paper which are in and of themselves a whole different effort, must cover a breadth of use-cases, architectures, data-types and data-shapes. Were we to superficially provide "sample" performance comparisons, or a sample code-comparison (same task done with different packages), we would risk misrepresenting other package's abilities, misleading the readers, or making incorrect claims about mdciao. We also stress that comparison to other software has been done, incidentally, to highlight mdciao's limitations with respect to other packages, and not the other way around.

1. **Methodology and Mathematical Explanation**: The paper lacks a comprehensive explanation of the methodologies and mathematical foundations that underpin each module within the "mdciao" package.

We thank the reviewer for this comment, and we have added a new paragraph explicitly declaring how the frequency computation takes place, given its central role to mdciao's function. This paragraph also serves to further highlight the explicit parametric dependence of contact-frequencies with the cutoff value more clearly, something that might have gone otherwise unnoticed.

In this spirit, we have also added a supplementary figure and a new paragraph explaining why the cutoff has been set to a default of 4.5 Angstrom, a point raised also by referee #3.

(Please note that because of this change, the figures needed to be updated, with slightly different contact-frequency reports)

As for mathematical foundations within each module of mdciao, we clarify that there aren't any more, except maybe with the exception of sequence-alignment algorithms to handle consensus labels, which fall outside the scope of the manuscript. Both in the manuscript and the documentation, we refer to BioPython and their documentation for sequence alignment.

Providing detailed descriptions and justifications for the chosen methods would greatly benefit readers, particularly those who wish to understand the technical aspects and validate the results produced by the tool.

We thank the reviewer for this comment and agree that, behind every choice made by mdciao there is a justification to be given. Given that residue-residue distances are central to mdciao, we have added some sentences detailing how this is done. As for other methodologies, they are mostly external to mdciao, i.e. provided by other modules, like the sequence alignment for consensus labels.

2. The "mdciao" package should provide a more detailed description of each module. The current paper does not sufficiently explain the context or the specific functionalities of each module. A comprehensive walkthrough of each module, including input requirements, processing steps, and output formats, would make the paper more informative and useful for potential users.

We thank the reviewer for this suggestion, and we now have added a new paragraph at the end of the section "Design-And-Implementation->API" which briefly describes the API and module structure, as well as a new Table (Table 2) outlining the specific intent of each module.

Beyond this, we kindly note that mdciao currently consists of 8 modules, each with between 3 and 20 public functions and classes, and each class with about a dozen methods. A *comprehensive walkthrough of each module* simply lies outside the scope of the manuscript. A scope, we note, that did not raise any issues with reviewers #2 and #3. We respectfully clarify that our intention is to showcase mdciao to the readers, not to *dissect* it for them, as it could potentially overwhelm

the type of reader for whom mdciao is trying to lower the entry barrier. Part of mdciao's value proposal is that non-experts do not even need to use the API but rather simple, one-shot CLI commands.

Annotated Figures and Tables: While the emphasis on producing annotated, production-ready figures and tables is commendable, the paper should include examples of these outputs. Demonstrating the tool's capability to generate high-quality visualizations would provide practical insights into its utility and effectiveness.

We thank the reviewer for his comment on our "commendable" effort, and kindly note that paper includes examples of these figures, tables, and outputs, both as inline figures and as separate pdf documents in the SI. These files are listed in the original submissions' file-inventory and are dynamically accessible for download from the unified document.

3. **Documentation and Tutorials**: The inclusion of a fully documented Python API and numerous examples and Jupyter Notebook tutorials is a strong point. However, the paper should highlight how these resources facilitate the integration of "mdciao" into existing workflows and support users in leveraging the tool's advanced features.

We thank the reviewer for their kind comments, we have indeed tried our best to make the manuscript figures also accessible for first-time users with the tutorials shipped with mdciao. With respect to API integration, Figure 5, panel b) cell [10] shows such an integration, when representative geometries extracted by mdciao are shown using nglview, without leaving the notebook. Another example is the usage of numpy for persistent storage of serialized mdciao objects in Figure 3, panel b) cell [12]. Both these instances are explicitly mentioned in the text as examples, together with other, more general operations like "clustering, time-series analysis, statistical modelling, plotting, formatting". We feel that integration with other APIs and packages is a general enough practice that does not need to be documented, as it is a hallmark feature of (I)Python/Notebook universe.

Other comments:

Line 35 readers would benefit if authors could provide some examples of these types of analyses and a link to the papers that performed them. This suggestion goes for all the analyses mentioned above. Additionally, it would be beneficial if authors could provide examples for each analysis and tool mentioned above, highlighting how these tools have contributed to the advancement of computational research. For example, have there been any groundbreaking findings that have transformed the way researchers have approached certain proteins?

In our introduction we have tried to accommodate a somewhat logical, step-by-step presentation of different analysis "levels" available to readers. Carrying out the above review, as asked by the referee, effectively amounts to a multi-decade chronology of a whole field, pinpointing what breakthrough was produced in what system with what software, and would be at risk of omitting crucial developments by simple reasons of succinctness. Thus, we respectfully stand by our

introduction, and assume readers are aware of these levels and methods, and place mdciao within that scheme.

Line 37-38 It's unclear what the authors are trying to convey here.

Thanks for pointing this out. Our intention was to separate Physics-based models, like MSMs, from other analysis levels (see above) and highlight that whereas approaches like contact-frequencies, PCA or clustering, are always possible to extract, MSMs are not, because they need large amounts of the right data, which might not be available because of the off-equilibrum nature of the simulation or because of the kinetic disconnectivity of the dataset. However, when attainable, these models provide a very comprehensive description of the system, and have predictive power over transition-paths, reaction-rates and equilibrium probabilities. They usually comprise a few states/geometries/conformations with distinct geometrical features and are independent of the dimensionality of the data, hence the mention to *compactness*. Without going into detail, we have rephrased for clarity as:

Provided that these models can be constructed with the available data, they offer a general representation of the MD data that is compact, captures the underlying model's physics, and is fully predictive.

Line 43–45 References to the paper that compiled these platoms would benefit the reader and future new users.

It is unclear to us what exactly is meant by this comment and would be grateful for clarification.

Line 48- 50 Provide an example of how different mdciao is from other Python modules. Could you provide one real example that correlates to well-known trajectory analysis tools?

line 52-60 This would be an excellent opportunity to highlight the direct differences from other available Python packages used for MD trajectory analysis. Currently, these statements span a wide range, and numerous Python packages provide all these features.

[we are answering the above paragraphs together]

The referee is correct in pointing out that indeed numerous python packages provide these features, each one doing so in different ways and expecting different scripting-capabilities from the user. This is a welcome hallmark of python modules and scripting across the board. And, as stated several times in the manuscript and in this letter, mdciao's value proposition is not "to do what other's cant", but simplify as much as possible "what others offer" to make it accessible (as stated in the manuscript's title). We note in passing that, technically, skilled-enough users will do a variety of things with bare-bone functionalities, and then any comparison would need to clarify things like "what technical skill is expected of MD analysists", a question that clearly interests us, but lies outside of the scope of the manuscript. Hence, instead of adding potentially misleading comparison statements, we respectfully stand by our choice to avoid direct, possibly incomplete

comparison to other modules, and have extensively argued above and elsewhere as to why we chose to do this.

The only place we felt confident in adding such a feature-comparison in a newly added sentence, at the end of the first paragraph of the discussion, where to the best of our knowledge mdciao is the only software including this feature:

Furthermore, for systems in which consensus nomenclature exists, analysis across different species, with very different primary sequences, can be streamlined without re-writing any code, for an arbitrary number of systems, ***a feature not presently implemented in any other software****.*

Line 64-67 Please describe briefly each panel, module, or analysis presented in Figure 1 or offered through MDCIAO. Authors should explain what these analyses are, why they matter, and how they differ from other Python trajectory analysis platforms. Additionally, provide an example comparison of mdcai and other md trajectory analyses, highlighting the usage time and computational resource requirements, and explain why using mdcaio is more beneficial for users compared to other packages.

Figure 1; label each panel in Figure 1 and provides a brief explanation of each window's appearance.

[we are answering the above paragraphs together]

Each panel in Figure 1 is labeled in the title-bar of the window-like pictogram. We believe the titles are descriptive in and of themselves, and are confident that readers are familiar with concepts like frequency-bar plots, violin-plots, distance distributions, flareplots, time-traces, contact matrices. We assume that these are all established enough not to be explained in detail, and note that the least frequent of them (the flareplot) is explained in the text in the results section.

Furthermore, all plots and outputs are shown again (except the time-dependent contact plots), in context, in Figures 2, 3, and 5, and explained in the text.

Table 1; Provide a concise explanation of the purpose of each tool, the expected output, and the process for interrupting the output. Please include pictures that illustrate the usage of each module in the supplementary section.

The concise explanation of each tool is provided by the two columns on each side of the tool's name, addressing its general nature (learning tool, pre-run tool, run tool, post-run tool) and its individual purpose. We note that, due to formatting issues, the last line of the table (the post-run tool mdc_compare.py) had been originally cut off. We note that, as the table itself notes, there is a tool, mdc_examples.py, which can be used to locally reproduce inputs and outputs for all other command-line-tools.

Section Example notebooks: Overall, this section of the paper needs **major work**. Please divide each case study separately and explain clearly. It would benefit the readers if the authors could provide comparisons with other tools.

Each notebook is a self-contained case study in itself, and conceptually represents one type of analysis one might do with mdciao. Each notebook has its own paragraph and thus we feel that readers will not be confused by this and note that the notebook figures are, precisely, separate case studies.

Performance and Scalability: It would be beneficial to include performance benchmarks that highlight the tool's efficiency and scalability. Information on how "mdciao" handles large datasets and its performance relative to other tools would be valuable for users dealing with extensive simulation data.

We thank the reviewer for this comment, and have added Supplementary Table 2, which summarizes the CPU time needed -on a multiprocessor desktop computer- to compute the frequencies shown in the newly added Supplementary Figure X. Again, we emphasize that mdciao's value proposition is not to out-perform more established and optimized libraries in raw performance numbers, and have respectfully refrained from comparing directly with other software.

Future Work and Development: The paper should outline potential areas for future development and improvement. This could include plans for additional features, enhancements to existing modules, or broader applications of the tool. Providing a roadmap for future updates can help engage the community and encourage contributions.

Community and Support: Highlighting the support available to users, such as forums, user groups, or dedicated support channels, can reassure potential users about the help they can expect when using the tool. Encouraging community involvement can also foster a collaborative environment for ongoing development.

[we are answering both paragraphs together]

We thank the referee for pointing this out and have added a statement "Community contributions are welcome, and we are committed to provide continuous support well be provided through https://github.com/gph82/mdciao/issues". Whereas no roadmap as such exists at the moment, a long list of known-issues and TODOs is available at https://github.com/gph82/mdciao?tab=readme-ov-file#todos.

In conclusion, while "mdciao" appears to be a promising tool for MD simulation analysis, the paper would benefit from a more detailed comparison with existing tools, a clearer explanation of methodologies, and a comprehensive description of each module. Addressing these points would significantly enhance the paper's value to the scientific community.

REVIEWER 2

Reviewer #2: Pérez-Hernández and Hildebrand report an elegant method to conveniently analyze Md trajectories with particular focus on inter-residue (or residue-ligand) contact mapping. It delivers a number of very useful, high resolution plots, allowing easily to compare systems (i.e. with different ligands, with mutations), particularly-but not limited to- on the cases of GPCRs, G-proteins ir kinases, as it adapts the standard nomenclature on those cases. A limitation that is conveniently outlined by the authors is the lack of decomposing on atom-residue mapping, probably more suited to drug-design projects, but the scope of mdciao is clearly stated to be on the protein-protein interactions (inter or intraprotein) and as such it is a very complete method.

The software installs easily, it is also available on-line, very well documented with a number of examples of e-g- jupyter-notebooks that can be used as a starting point for novel users.

I have only a few minor corrections of typos or grammar as follows:

- line 41- "MDanalysis, The API" replace comma by dot

- line 153 - "a growing list works " probably "a growing list OF works (studies?)"

We thank the reviewer for their kind words and are happy they found no issue with the manuscript. Their corrections have been implemented in the revised text.

REVIEWER 3

Reviewer #3: The manuscript “mdciao: Accessible Analysis and Visualization of Molecular Dynamics Simulation Data” by Perez-Hernandez and Hildebrand presents a software, actually command line tools and a python API, for analysis and representation of molecular dynamics (MD) simulation data. While many programmes exist to analyse and/or visualise MD data, including python modules rich in functionality, the authors claim to fill a gap by catering to non-expert users (with production-ready tables and figures) and expert users alike (by full flexibility to use the API in their python workflow).

The software is indeed useful and the description and examples provided in the present manuscript provide a good overview and introduction into its functionality, in particular thanks to the access to worked examples.

We thank the reviewer for their kind words, and are happy our point about “filling a gap” came across clearly. We specifically want to thank them also for prompting us to reexamine the way in which the hard-cutoff is presented in the manuscript and in the software. It ultimately lead to a more explicit declaration of the cutoff vs frequency relation in the text and, importantly, to the fine-tuning of mdciao’s default parameter. See below for more details.

However, a few things need to be improved in the manuscript:

In the list of the added values, the authors mention, among others, “residue-residue contact-frequencies with hard cutoffs” as an easy and universal metric. However, it is not fully explained why this is desirable as the most important , if not only (?) metric, in the first place.

The authors discuss that such contacts defined via a hard cut-off are preferable over more fine-tuned parameters and should therefore be transferable. Given the importance of the contacts and the corresponding cut-off, it should be detailed more in the methods section of the manuscript. That is which of the “typically 3.5-4.5A” is chosen (say, as user-friendly default)? To which atoms does this distance cutoff apply? To all atoms of two residues in question? Only heavy atoms? The nearest two?

We sincerely thank this reviewer for this comment, which led us to an effort to clarify, even more, what we mean with transferable and why we think that is a good quality.

Prompted by this comment, we carried out a set of sample computations on a diverse set of publicly available MD simulations. After compiling heavy-atom—heavy-atom residue-residue distances, we scanned the effect of different cutoffs on reference frequencies. The result of these cursory parameter exploration ultimately led us to change the default cutoff value to 4.5A, since it performed better across a variety of situations. Three new Supplementary Figures, and a (in our opinion) a better paragraph clarifying what the “single” cutoff is trying to achieve resulted from this comment, so we again thank the reviewer for their comment.

Furthermore, the authors claim to “special care on user-friendliness, documentation (inline and online) and tutorials”. The first can be tested by only using the software – and appears to be achieved for this reviewer. Documentation and tutorials are provided as example notebooks, or learning command line tools, and these are highly appreciated.

We thank the reviewer for their kind words.

The link to documentation and tutorials should be placed more prominently in the manuscript, perhaps even in the abstract close to the link to the github repository.

We thank the reviewer for their remark, and now we reference mdciao.org in the abstract.
