# Supplementary material for: mdciao: Accessible Analysis and Visualization of Molecular Dynamics Simulation Data
Source: PLoS Comput Biol. 2025 Apr 21;21(4):e1012837. doi: 10.1371/journal.pcbi.1012837 (PMC12011235; doi:10.1371/journal.pcbi.1012837)
Supplement: Attachment — Submitted filename: Review of Mdciao.docx [file pcbi.1012837.s009.docx]

Review of “mdciao: An Open-Source Tool for Molecular Dynamics Simulation Analysis”

The paper presents “mdciao,” an open-source command line tool and Python API designed for the analysis and representation of molecular dynamics (MD) simulation data. While the tool offers a user-friendly interface and a range of pre-packaged solutions for both non-expert and expert users, several critical areas require improvement to enhance the paper’s clarity and comprehensiveness.

Comparison with Existing Tools: One significant omission in the paper is a comparison of “mdciao” with other existing tools in the field. A detailed comparison would provide readers with a clearer understanding of the unique advantages and potential limitations of “mdciao” relative to other available software. This comparison should include performance metrics, type of use, and the range of functionalities offered.

Methodology and Mathematical Explanation: The paper lacks a comprehensive explanation of the methodologies and mathematical foundations that underpin each module within the "mdciao" package. Providing detailed descriptions and justifications for the chosen methods would greatly benefit readers, particularly those who wish to understand the technical aspects and validate the results produced by the tool.

The "mdciao" package should provide a more detailed description of each module. The current paper does not sufficiently explain the context or the specific functionalities of each module. A comprehensive walkthrough of each module, including input requirements, processing steps, and output formats, would make the paper more informative and useful for potential users.

Annotated Figures and Tables: While the emphasis on producing annotated, production-ready figures and tables is commendable, the paper should include examples of these outputs. Demonstrating the tool’s capability to generate high-quality visualizations would provide practical insights into its utility and effectiveness.

Documentation and Tutorials: The inclusion of a fully documented Python API and numerous examples and Jupyter Notebook tutorials is a strong point. However, the paper should highlight how these resources facilitate the integration of “mdciao” into existing workflows and support users in leveraging the tool’s advanced features.

Other comments:

Line 35 readers would benefit if authors could provide some examples of these types of analyses and a link to the papers that performed them. This suggestion goes for all the analyses mentioned above. Additionally, it would be beneficial if authors could provide examples for each analysis and tool mentioned above, highlighting how these tools have contributed to the advancement of computational research. For example, have there been any groundbreaking findings that have transformed the way researchers have approached certain proteins?

Line 37-38 It's unclear what the authors are trying to convey here.

Line 43–45 References to the paper that compiled these platoms would benefit the reader and future new users.

Line 48- 50 Provide an example of how different mdciao is from other Python modules. Could you provide one real example that correlates to well-known trajectory analysis tools?

line 52-60  This would be an excellent opportunity to highlight the direct differences from other available Python packages used for MD trajectory analysis. Currently, these statements span a wide range, and numerous Python packages provide all these features.

Line 64-67 Please describe briefly each panel, module, or analysis presented in Figure 1 or offered through MDCIAO. Authors should explain what these analyses are, why they matter, and how they differ from other Python trajectory analysis platforms. Additionally, provide an example comparison of mdcai and other md trajectory analyses, highlighting the usage time and computational resource requirements, and explain why using mdcaio is more beneficial for users compared to other packages.

Figure 1; label each panel in Figure 1 and provides a brief explanation of each window's appearance.

Table 1; Provide a concise explanation of the purpose of each tool, the expected output, and the process for interrupting the output. Please include pictures that illustrate the usage of each module in the supplementary section.

Section Example notebooks: Overall, this section of the paper needs major work. Please divide each case study separately and explain clearly. It would benefit the readers if the authors could provide comparisons with other tools.

Performance and Scalability: It would be beneficial to include performance benchmarks that highlight the tool’s efficiency and scalability. Information on how “mdciao” handles large datasets and its performance relative to other tools would be valuable for users dealing with extensive simulation data.

Future Work and Development: The paper should outline potential areas for future development and improvement. This could include plans for additional features, enhancements to existing modules, or broader applications of the tool. Providing a roadmap for future updates can help engage the community and encourage contributions.

Community and Support: Highlighting the support available to users, such as forums, user groups, or dedicated support channels, can reassure potential users about the help they can expect when using the tool. Encouraging community involvement can also foster a collaborative environment for ongoing development.

In conclusion, while “mdciao” appears to be a promising tool for MD simulation analysis, the paper would benefit from a more detailed comparison with existing tools, a clearer explanation of methodologies, and a comprehensive description of each module. Addressing these points would significantly enhance the paper’s value to the scientific community.
